# Piezoelectric Biomaterials for Bone Regeneration: Roadmap from Dipole to Osteogenesis

**DOI:** 10.1002/advs.202414969

**Published:** 2025-06-19

**Authors:** Xiyao Ni, Yufei Cui, Mojtaba Salehi, Mui Ling Sharon Nai, Kun Zhou, Cian Vyas, Boyang Huang, Paulo Bartolo

**Affiliations:** ^1^ Singapore Centre for 3D Printing School of Mechanical and Aerospace Engineering Nanyang Technological University Singapore 639798 Singapore; ^2^ Additive Manufacturing Division Singapore Institute of Manufacturing Technology (SIMTech) Agency for Science Technology and Research (A*STAR) 5 Cleantech Loop Singapore 636732 Singapore

**Keywords:** biomaterial, bone tissue engineering, molecular dipole, osteogenesis, piezoelectricity

## Abstract

Piezoelectric biomaterials convert mechanical energy into electrical charges, making them promising candidates for bone tissue engineering by restoring and modulating the electrophysiological microenvironment. This review explores the development of piezoelectric biomaterials by focusing on their molecular origins, particularly dipoles, and how their type, source, and spatial arrangement influence macroscopic electromechanical coupling. Beyond intrinsic origins, the concept of pseudo‐piezoelectricity driven by extrinsic factors is introduced to highlight alternative approaches for piezoelectric biomaterial design. Techniques to engineer dipoles and modulate piezoelectric properties for the regulation of osteogenesis are discussed. Particular attention is given to the correlation between piezoelectricity and osteogenesis at distinct phases of bone regeneration. Finally, current challenges in molecular understanding and biofabrication of piezoelectric bone scaffolds are highlighted, along with potential future research directions.

## Graphical abstract

Piezoelectric biomaterials convert mechanical forces into electrical signals, offering novel strategies to restore and modulate bone microenvironments for tissue engineering. This review examines molecular dipole origins, spatial arrangements, and pseudo‐piezoelectric mechanisms and highlights dipole‐engineering techniques for osteogenesis regulation. Correlations between electromechanical coupling and bone regeneration phases are analyzed, and fabrication challenges and future research directions are proposed.

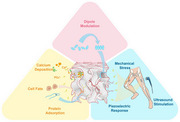

## 1 Introduction

Bone is a remarkable tissue responsible for various bodily functions, including the production of red and white blood cells, the protection of internal organs, and enabling locomotion. The tissue undergoes different stages during its lifespan, including bone development, bone remodelling, and fracture‐healing in response to non‐critical injuries. Molecular and cellular events are crucial to facilitate normal tissue function. However, the importance of dynamic changes in the electrophysiological microenvironment, i.e., the membrane potential imbalance produced via ion channels and gap junctions, is crucial and typically underappreciated^[^ ^1^, ^2^, ^3^, ^4^ ^]^ This becomes especially relevant during fracture healing, which involves multiple phases, including vascular breakdown, hematoma formation, inflammation, revascularization, cell migration, proliferation, differentiation, biomineralization, and bone remodelling.^[^ ^5^, ^6^, ^7^ ^]^ The electrophysiological microenvironment can be restored via the regeneration of periosteum‐like tissue following non‐critical bone injuries, which supports osteogenesis through the modulation of cell attachment, proliferation, differentiation, and macrophage polarization.^[^ ^8^, ^9^, ^10^ ^]^

However, in extremes of bone healing, i.e., critical bone defects such as trauma, tumour removal, and infected necrosis, the spontaneous fracture‐healing is no longer possible.^[^ ^11^, ^12^ ^]^ Typically, the gold standard for treating such defects has been bone grafting using autologous or allogenic grafts, though this approach faces major limitations, including restricted tissue volume and secondary trauma at the harvesting site.^[^ ^13^ ^]^ Therefore, tissue‐engineered constructs have been developed as an alternative to specifically guide and promote tissue regeneration.^[^ ^14^, ^15^, ^16^, ^17^ ^]^ Within the field of bone tissue engineering (BTE), the use of electroactive biomaterials (e.g., conductive and piezoelectric), has gained attention for modulating the bioelectrical microenvironment of bone tissue.^[^ ^4^ ^]^

The piezoelectric effect, defined as the conversion between electrical and mechanical energy, was first demonstrated by the brothers Pierre and Jacques Curie in 1880 in quartz and Rochelle salt. The term is derived from the Ancient Greek “piezo”, meaning “to press”.^[^ ^18^ ^]^ As early as 1957, the human femur bone was demonstrated to have piezoelectric properties similar to those of wood,^[^ ^19^ ^]^ suggesting its capability of converting mechanical strain into electrical polarization.^[^ ^20^ ^]^ The piezoelectricity in bone was believed to originate from the non‐centrosymmetric nature of collagen fibers. Further studies highlighted the role of hydroxyapatite (HA) present in bone tissue in maintaining this non‐centrosymmetry within collagen fibers in the presence of moisture.^[^ ^21^ ^]^ In 1975, Reinish and Nowick reported that the principle piezoelectric property of the femur is the shear piezoelectric coefficient *d*_14_ ≈ 7 pC/N.^[^ ^22^ ^]^ In 2004, Halperin et al.^[^ ^23^ ^]^ employed the piezoresponse force microscope (PFM) and revealed that the femur also exhibits a longitudinal piezoelectric coefficient *d*_33_ = 7.66–8.72 pC/N. Inspired by the piezoelectricity of native bone tissue, the concept of piezoelectric scaffolds, i.e., self‐powered electroactive scaffolds, has gained attention for their ability to convert mechanical strain from daily motion or other non‐invasive forms (e.g., ultrasonic waves), into electrical stimulation, thus recapitulating the endogenous bioelectrical microenvironment and promoting bone regeneration.^[^ ^24^ ^]^ A prerequisite for such materials is biocompatibility, which excludes lead‐based piezoelectric ceramics due to their cytotoxicity. Subsequently, piezoelectric biomaterials commonly utilized for BTE are mainly derived from a subset of inorganic (e.g., barium titanate)^[^ ^25^, ^26^, ^27^ ^]^ and organic (e.g., polyvinylidene fluoride) materials.^[^ ^28^, ^29^, ^30^, ^31^ ^]^

As the number of studies on piezoelectric materials for BTE continues to grow, reviews have discussed the potential of piezoelectric materials from distinct perspectives. For example, ceramic,^[^ ^32^ ^]^ polymeric‐based,^[^ ^32^, ^33^ ^]^ and natural^[^ ^34^ ^]^ piezoelectric biomaterials and their cellular interactions have been highlighted. Furthermore, Khare et al.^[^ ^35^ ^]^ discussed processing‐related challenges and the relationship between piezoelectric properties, especially specific crystalline phases, and their osteogenic promotive effect. Alternatively, Chen et al.^[^ ^36^ ^]^ emphasized the importance of additive manufacturing in the development of the next generation of piezoelectric bone scaffolds. Moreover, comprehensive discussions around the design and role of piezoelectric materials in a wider range of applications, including tissue engineering, sensors, actuators, and therapeutic treatments, can be found in many reviews.^[^ ^24^, ^37^, ^38^, ^39^, ^40^ ^]^ However, there is limited discussion on the relationship between the electromechanical origins of piezoelectricity, i.e., the individual dipoles, their modulation during fabrication, and their role in osteogenesis.

Therefore, this review presents a roadmap from the piezoelectric origins to osteogenesis. Specifically, the classification of dipoles serves as a foundation for summarising and discussing the macroscopic piezoelectric behavior in various assembled conformations. Additionally, the extrinsic origins, i.e., charged voids and diffusing ions, of pseudo‐piezoelectricity are presented. Subsequently, the engineering of the dipoles with a view to modulate the macroscopic piezoelectric performance is discussed. Finally, the relationship between the piezoelectric parameters and the key phases of the cell‐biomaterial interactions (e.g., protein adsorption, calcium deposition, cell fate decision, and signalling pathways) that impact osteogenesis and bone regeneration is addressed.

## 2 Intrinsic Origins of Piezoelectric Biomaterials

**Figure 1:**
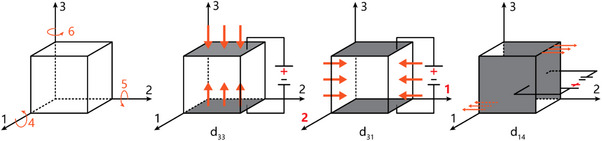
Three‐axis coordinate system for the schematic representation of piezoelectric responses characterized by constants *d*_31_, *d*_32_, and *d*_14_. Orange arrows denote the direction of applied stress, and the surfaces that generate a charge are marked in grey.

In general, piezoelectric materials are characterized by their capability to generate electrical polarization upon exposure to mechanical stimuli and vice versa. They undergo mechanical strain when energized by exogenous electrical stimuli.^[^ ^41^, ^42^ ^]^ To characterize the monolithic piezoelectric response, the piezoelectric coefficient *d_ij_* is employed to describe the proportional relationship between the generated displacement *D_i_* in polarization and driving stress *T_j_*:^[^ ^43^, ^44^ ^]^

(1) $$d_{ij}=\frac{\partial D_{i}}{\partial T_{j}}=\left(\begin{array}{cccccc} 0 & 0 & 0 & d_{14} & d_{15} & 0\\ 0 & 0 & 0 & d_{24} & d_{25} & 0\\ d_{31} & d_{32} & d_{33} & 0 & 0 & d_{36} \end{array}\right)$$

where *i* denotes the direction of the generated electric field in three axes, taking values of 1, 2, or 3; *j* represents the direction of applied stress varying from 1 to 6,^[^ ^45^ ^]^ wherein1–3 and 4–6 represent the direction of the normal stress parallel and the shear stress perpendicular to axes 1—3, respectively.^[^ ^46^ ^]^ **Figure** **1** shows the three‐axis coordinate system and the commonly utilized piezoelectric responses such as the normal effects with constants *d*_33_ or *d*_31_, and shear effect with constant *d*_14_. The longitudinal piezoelectric coefficient *d*_33_ is related to the generated electrical polarization along the same direction as the applied stress. The transverse piezoelectric coefficient *d*_31_ describes the generation of polarization in axis three activated by stress in axis one, while the shear piezoelectric coefficient *d*_14_ quantifies the electrical polarization in axis one induced by the shear deformation within the 2–3 plane.^[^ ^47^ ^]^ The absolute values of these corresponding piezoelectric coefficients determine the amplitude of the transient charge fluctuations when subjected to external mechanical stimuli such as periodic loading and ultrasound (US).^[^ ^48^ ^]^

**Figure 2:**
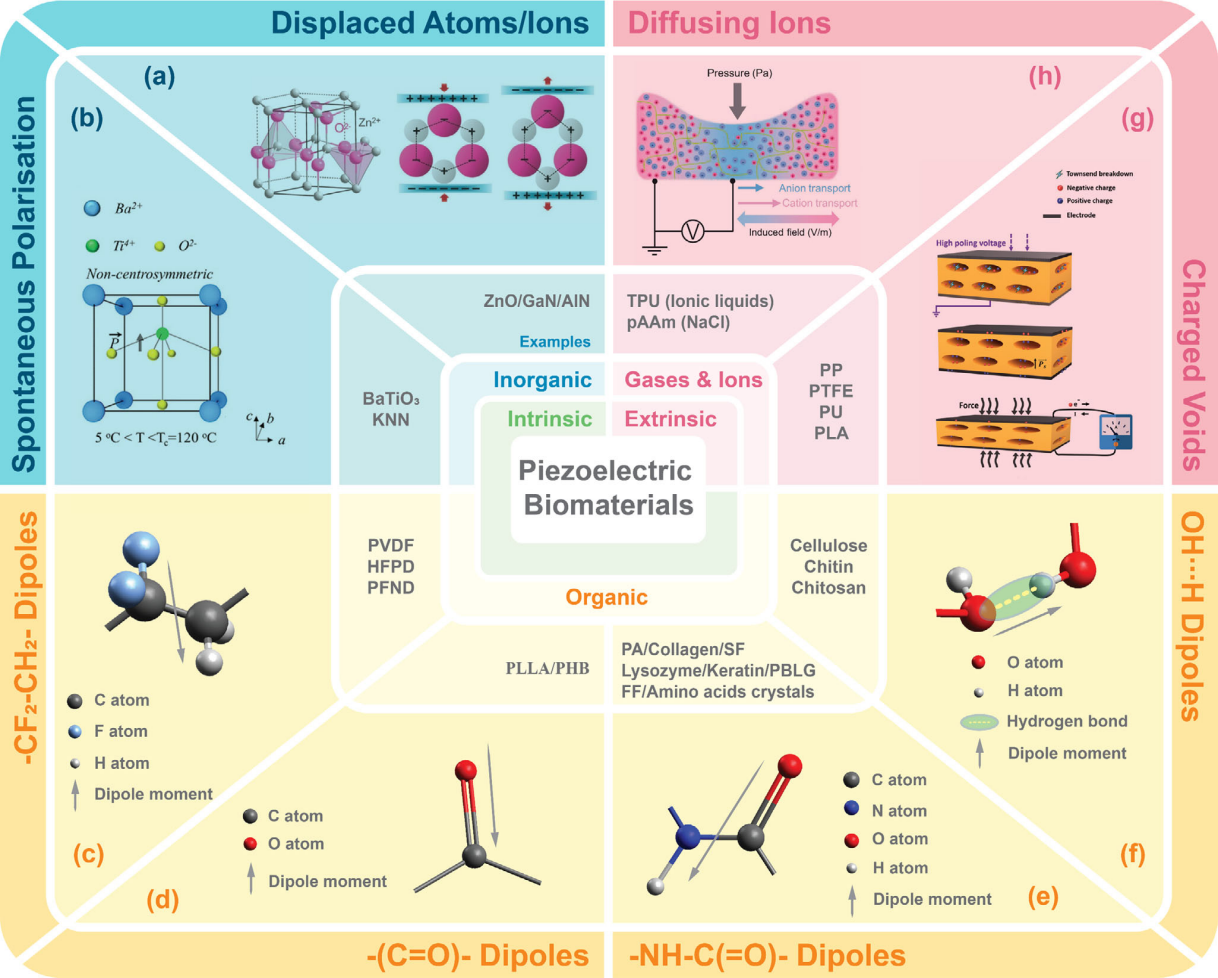
Electromechanical origins of inorganic/organic piezoelectric biomaterials and pseudo‐piezoelectric matrix. a) Atoms/ions displacement in the lattice and corresponding polarization of ZnO. Reproduced under the terms of the CC BY 4.0 license.^[^ ^54^ ^]^ Copyright 2017, WILEY‐VCH Verlag GmbH & Co. KGaA. b) Non‐zero dipole moment from spontaneous polarization induced by a deviating atom from the lattice center in BT. Reproduced under the terms of the CC BY‐NC 4.0 license.^[^ ^60^ ^]^ Copyright 2020, Wiley‐VCH GmbH. Schematic illustration of dipole moment generated in (c), [─CF_2_─CH_2_─] (d) [─(C═O)─] dipoles, e) [─NH─C(═O)─] dipoles and f) intra‐/intermolecular OH∙∙∙O hydrogen bonds. g) Townsend breakdown of gas in voids by external electric field resulting in a dielectric barrier discharge (DBDs) and electromechanical response of charged voids within the material matrix. Reproduced with permission.^[^ ^61^ ^]^ Copyright 2019, Elsevier. h) Ion diffusion under localized mechanical loading and corresponding electrical potential signals generated between adjacent regions. Reproduced with permission.^[^ ^62^ ^]^ Copyright 2022, The American Association for the Advancement of Science.

As illustrated in **Figure** **2**, the derivation of piezoelectricity primarily stems from the inherent characteristics of the piezoelectric materials. Nevertheless, in some cases, piezoelectric‐like responses can be excited paradoxically using non‐piezoelectric materials. In this section, the origin of piezoelectricity is presented from the perspective of the lattice structural arrangement/displacement (inorganic materials) and the class of dipoles (organic materials), whilst the pseudo‐piezoelectric response is discussed in the following section (non‐polar biomaterials).

### 2.1 Inorganic Piezoelectric Biomaterials

Typically, in inorganic piezoelectric biomaterials such as zinc oxide (ZnO),^[^ ^49^ ^]^ gallium nitride (GaN),^[^ ^50^ ^]^ and aluminium nitride (AlN),^[^ ^51^ ^]^ electrical polarization arises from fluctuations in the net electric dipole moment. This phenomenon is attributed to the displacement of ions and atoms within the crystal lattice, induced by applied mechanical stimuli, see Figure 2a.^[^ ^52^, ^53^, ^54^ ^]^ Therefore, a certain degree of non‐centrosymmetry in the crystal lattice is essential for this class of material. Otherwise, the electric dipole moment and its corresponding inversion will be null, even in the presence of strain, resulting in a zero net electric dipole moment.^[^ ^47^ ^]^ Nevertheless, in distinctive cases such as barium titanate (BT)^[^ ^55^ ^]^ and lead‐free potassium sodium niobate (KNN)^[^ ^56^ ^]^ centrosymmetry vanishes due to an additional central atom deviating from the inversion centre of the unit cell. This deviation leads to the formation of a spontaneous non‐zero net dipole moment $\vec{P}$ inside the unit cell (Figure 2b).^[^ ^57^, ^58^ ^]^ When exposed to a large external electric field, the spontaneous $\vec{P}$ reorients parallel to the direction of the external field and does not reverse upon the removal of that field, inducing the ferroelectric phenomenon. The external field‐induced reorientation of the spontaneous $\vec{P}$ is understood as a poling process.^[^ ^30^ ^]^ Piezoelectric materials with spontaneous $\vec{P}$ are therefore further categorized into a ferroelectric material subset. Untreated bulk ferroelectric materials may contain randomly oriented crystal domains, with the spontaneous $\vec{P}$ in their crystallographic orientation to minimize the electrostatic energy, namely ferroelectric domains.^[^ ^59^ ^]^ As the strength of the electrical poling increases, an increasing fraction of ferroelectric domains switch to the poling direction.^[^ ^47^, ^59^ ^]^ Upon removal of the poling field, the treated ferroelectric domains undergo only minimal reversal or reorientation, and the aligned $\vec{P}$ leads to a non‐zero polarization of the bulk material, known as remnant polarization (*P*_r_).^[^ ^59^ ^]^

In biomedical applications, *P*_r_ as a unique bulk characteristic in ferroelectric materials is intimately associated with the resting material surface charge, which plays a vital role in regulating protein adsorption, cell adhesion, and subsequent cell behavior at the cell‐material interface.^[^ ^63^ ^]^ It is important to note that the surface charges mentioned in this context belong to the class of bound charges (current non‐conductible), rather than free charges that can travel through the volume of the material (current conductible).^[^ ^64^ ^]^ As opposed to the resting material surface charge, the fluctuating surface charge due to polarization triggered by an external stimulus is called the transient surface charge.^[^ ^65^ ^]^ For a given mode and magnitude of external stimuli, the amplitude of the fluctuation in transient surface charge strongly depends on the efficiency of electromechanical conversion.^[^ ^66^ ^]^ Thus, aligned ferroelectric domains have a higher electromechanical conversion. On the contrary, in non‐ferrous piezoelectric materials without spontaneous $\vec{P}$ (i.e., AlN, GaN, and ZnO), once crystal deposition is completed during material preparation, the internal crystal arrangement and bulk piezoelectric properties remain unchanged.^[^ ^37^ ^]^ This stability is highly desirable for industrial applications such as capacitors, sensors, transducers, nano‐generators, and actuators with superior electromechanical conversion capability. However, the correlation between improved osteogenesis and ever‐increasing resting/transient surface charge performance is inconclusive, and an in‐depth discussion is presented in Section 5.

### 2.2 Organic Piezoelectric Biomaterials

Organic piezoelectric biomaterials are widely explored in tissue engineering, especially BTE, due to their simple production, ductility, flexibility, fracture toughness, biocompatibility, and biodegradability.^[^ ^38^ ^]^ Similar to inorganic piezoelectric materials, the bulk polarization of an organic piezoelectric biomaterial is the vector sum of the dipole moments (net dipole moments) within the material matrix. However, these dipole moments are believed to be the measure of the polarity of intra‐/intermolecular polar bonds formed by atoms with different electronegativities, i.e., molecular dipoles.^[^ ^67^ ^]^ Compared to the linear displacement in highly ordered ion‐bonded lattices of inorganic piezoelectric biomaterials, the piezoelectric response of organic piezoelectric biomaterials is mainly attributed to the molecular chain conformation and reorientation of molecular dipoles under mechanical stress.

Non‐centrosymmetry at the molecular level remains essential for achieving non‐zero piezoelectric tensors. The complex spatial conformation of the non‐centrosymmetric molecules, for example, the alternating distribution of disordered amorphous and ordered crystalline regions in semicrystalline polymers, impedes the investigation of the bulk piezoelectric response in organic materials.^[^ ^68^ ^]^ In addition to the vastly different molecular chain conformations, the propagation mode of the applied stress/strain varies greatly between amorphous and crystalline regions.^[^ ^69^ ^]^ Typically, the discussion of piezoelectric properties is framed by the origin of the biomaterial, i.e., natural (e.g., polysaccharides and proteins) and synthetic,^[^ ^70^, ^71^ ^]^ or the structural conformation of molecular chains, i.e., crystalline, semi‐crystalline, and amorphous.^[^ ^68^, ^72^ ^]^ However, none of the reviews are exclusively from the perspective of the molecular dipole. Herein, the origin of molecular dipoles and their spatial arrangement from the microscopic to macroscopic scale are presented, to provide an intuitive understanding of the electromechanical conversion in organic piezoelectric biomaterials.

#### 2.2.1 [─CF_2_─CH_2_─] Dipole

**Figure 3:**
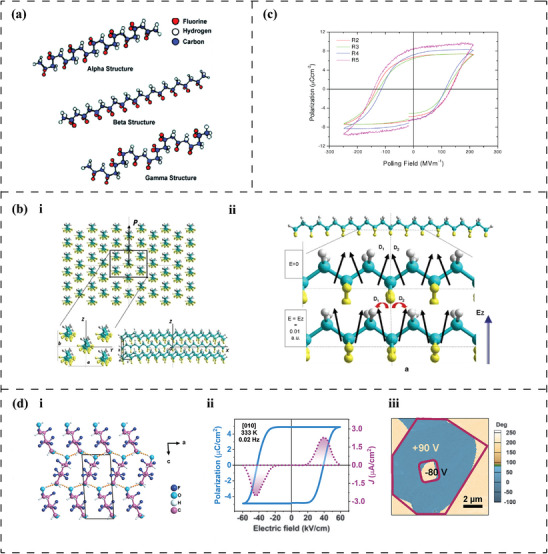
Piezoelectricity originates from [─CF_2_─CH_2_─] dipoles. a) Chain conformation and atomic arrangement in different PVDF crystalline phases. Reproduced under the terms of the CC BY 4.0 licence.^[^ ^79^ ^]^ Copyright 2018, MDPI. Molecular schematics of (b_i_) the hexagonal packing of PVDF chains in *β*‐phase and the net dipole moment $\vec{P}$ as the consequence of the superposition of all [─CF_2_─CH_2_─] dipoles (yellow: fluorine, grey: hydrogen, and cyan: carbon), and (b_ii_) the rotation of [─CF_2_─CH_2_─] dipoles under an applied electric field. Reproduced with permission.^[^ ^80^ ^]^ Copyright 2013, Springer Nature. c) Polarization‐electrical field hysteresis loops of PVDF, where *β* phase increases with increasing stretching ratio (*R* = 2–5). Reproduced with permission.^[^ ^81^ ^]^ Copyright 2010, IOP Publishing. Molecular model of (d_i_) the asymmetric layered stacking of HFPD chains under the force of hydrogen bonding (yellow dashed line), and (d_ii_) polarization‐electric field loop and (d_iii_) phase image of HFPD in the form of a single crystal and thin film, respectively. Reproduced with permission.^[^ ^82^ ^]^ Copyright 2024, The American Association for the Advancement of Science.

Molecular dipole moments originating from F─C and C─H bonds can be found in the polar difluoromethylene group (─CF_2_) and methylene group (─CH_2_) of vinylidene fluoride. [─CF_2_─CH_2_─] A popular piezoelectric and biocompatible semi‐crystalline polymer, poly(vinylidene fluoride) (PVDF), and its copolymers (e.g., poly(vinylidene fluoride‐co‐trifluoroethylene) (PVDF‐TrFE)), are comprised of the [─CF_2_─CH_2_─] monomer and exhibit a comparable piezoelectric constant to that of piezoelectric ceramics.^[^ ^67^, ^73^, ^74^ ^]^ In each [─CF_2_─CH_2_─] unit, the oppositely arranged fluorine and hydrogen atoms (electronegative pairs) possess an extraordinary dipole moment (Figure 2c) of two Debye, i.e., [─CF_2_─CH_2_─] units can be viewed as intrachain dipoles.^[^ ^73^, ^75^ ^]^ If a highly ordered arrangement of these dipoles is achieved, it is conceivable that PVDF would exhibit macroscopic polarity and piezoelectric properties. The [─CF_2_─CH_2_─] units arrange into five distinct crystalline phases (*α, β, γ, δ*, and *ε*) or a randomly oriented amorphous region. Among them, the *β* crystalline phase exhibits a unique hexagonal packing of all‐trans (TTTT) planar zigzag chain conformation. Such uniform zigzag alignment of the [─CF_2_─CH_2_─]_n_ chains, allows the consistent arrangement of all [─CF_2_─CH_2_─] dipoles in a parallel manner.^[^ ^76^, ^77^ ^]^ Therefore, all fluorine atoms are arranged on the same side of the PVDF chain, while all hydrogen atoms are arranged on the other side (**Figure** **3**a), which in turn yields an effective superposition of dipole moments perpendicular to the [─CF_2_─CH_2_─] chain direction (Figure 3b_i_).^[^ ^78^, ^79^, ^80^ ^]^

The highly ordered *β* crystalline arrangement of [─CF_2_─CH_2_─] dipoles provides PVDF with remarkable electromechanical conversion capabilities. Bystrov et al.^[^ ^80^ ^]^ predicted the rotation of [─CF_2_─CH_2_─] dipoles on PVDF chains under an applied field using First‐principles calculations and Molecular Mechanics methods (Figure 3b_ii_). This rotation induces changes in the height and length of the polymer chains, resulting in the deformation of the chain skeleton. Computational interrogation of this electrostriction model yielded a longitudinal and transverse piezoelectric coefficient of *d*_33_ ≈ −33.5 to −38.5 pC/N (for dielectric constant ε = 10) and *d*_31_ ≈ 15.5 pC/N, respectively, conforming with experimental values. In addition, the switchable [─CF_2_─CH_2_─] dipoles under a poling field confer inherent ferroelectricity to the PVDF *β* phase. In a subsequent study, the four‐chain PVDF model revealed that [─CF_2_─CH_2_─] dipoles can direct the PVDF chains to exhibit homogeneous switching above the critical field *E* = *E*_c_ (coercive field).^[^ ^73^ ^]^ Furthermore, Gomes et al.^[^ ^81^ ^]^ demonstrated the positive correlation between the fraction of switching [─CF_2_─CH_2_─] dipoles within the *β* phase and critical parameters such as coercive field (*E_c_*), saturated polarization (*P*_s_), and *P*_r_ (Figure 3c). Therefore, the unique arrangement of [─CF_2_─CH_2_─] dipoles within the *β* phase can be identified as the primary origin of the piezoelectric properties of PVDF and its copolymers.

Although PVDF and its copolymers are chemically inert, biocompatible, and highly piezoelectric, their non‐biodegradability limits their application in bioabsorbable implants.^[^ ^83^ ^]^ However, Zhang et al.^[^ ^82^ ^]^ discovered the superior piezoelectric properties (*d*_33_ ≈ 138 pC/N) in a molecular crystal HOCH_2_(CF_2_)_3_CH_2_OH [2,2,3,3,4,4‐hexafluoropentane‐1,5‐diol (HFPD), which exhibits both biocompatibility and biodegradability. In its repeating molecular chains, two [─CF_2_─CH_2_─] dipoles are linked by an oppositely oriented [─CF_2_─] unit, resulting in a non‐zero spontaneous dipole moment perpendicular to the chain direction (Figure 3d_i_). The hydrogen bonds formed between the terminal hydroxyl groups induce the asymmetrically layered stacking of repeating units without cancelling the dipole moments. Moreover, HFPD also exhibits ferroelectricity in the form of either a single crystal or a film (Figure 3d_ii_,d_iii_), i.e., the rotatability of dipoles under a switching electric field. Furthermore, Ai et al.^[^ ^84^ ^]^ utilised the strategy of modifying 1H,1H,9H,9H‐perfluoro‐1,9‐nonanediol (PFND) with [─CH_2_OH] as anchoring terminal and produced molecular crystal HOCH_2_(CF_2_)7CH_2_OH with odd‐numbered repeating [─CF_2_─] units. Similar to HFPD, the hydrogen bonding network imparts an asymmetric stacking conformation to the PFND chains and a non‐zero net polarization to the PFND crystals. Consequently, the PFND crystal exhibits a moderate piezoelectric coefficient *d*_33_ ≈ 6 pC/N. Additionally, promising in vitro biocompatibility as well as in vivo biosafety and biodegradability were demonstrated.^[^ ^84^ ^]^ These developments in [─CF_2_─CH_2_─] based biomaterials, such as HFPD and PFND, broadens the library of piezoelectric materials available for BTE.

#### 2.2.2 [─(C═O)─] Dipole

The molecular dipoles originating from the C═O double bond are prevalent in ester groups, [R─(C═O)─O─R’] playing a crucial role in defining the piezoelectric properties of synthetic polymers such as poly(L‐lactic acid) (PLLA) and polyhydroxy butyrate (PHB). These [─(C═O)─] dipoles (Figure 2d) exhibit a considerable dipole moment, typically around 1.8 Debye, directed at an angle of 123° from the R─(C═O) axis.^[^ ^85^ ^]^ The origin of this pronounced dipole moment lies in the substantial difference in electronegativity between the carbon and oxygen atoms.

**Figure 4:**
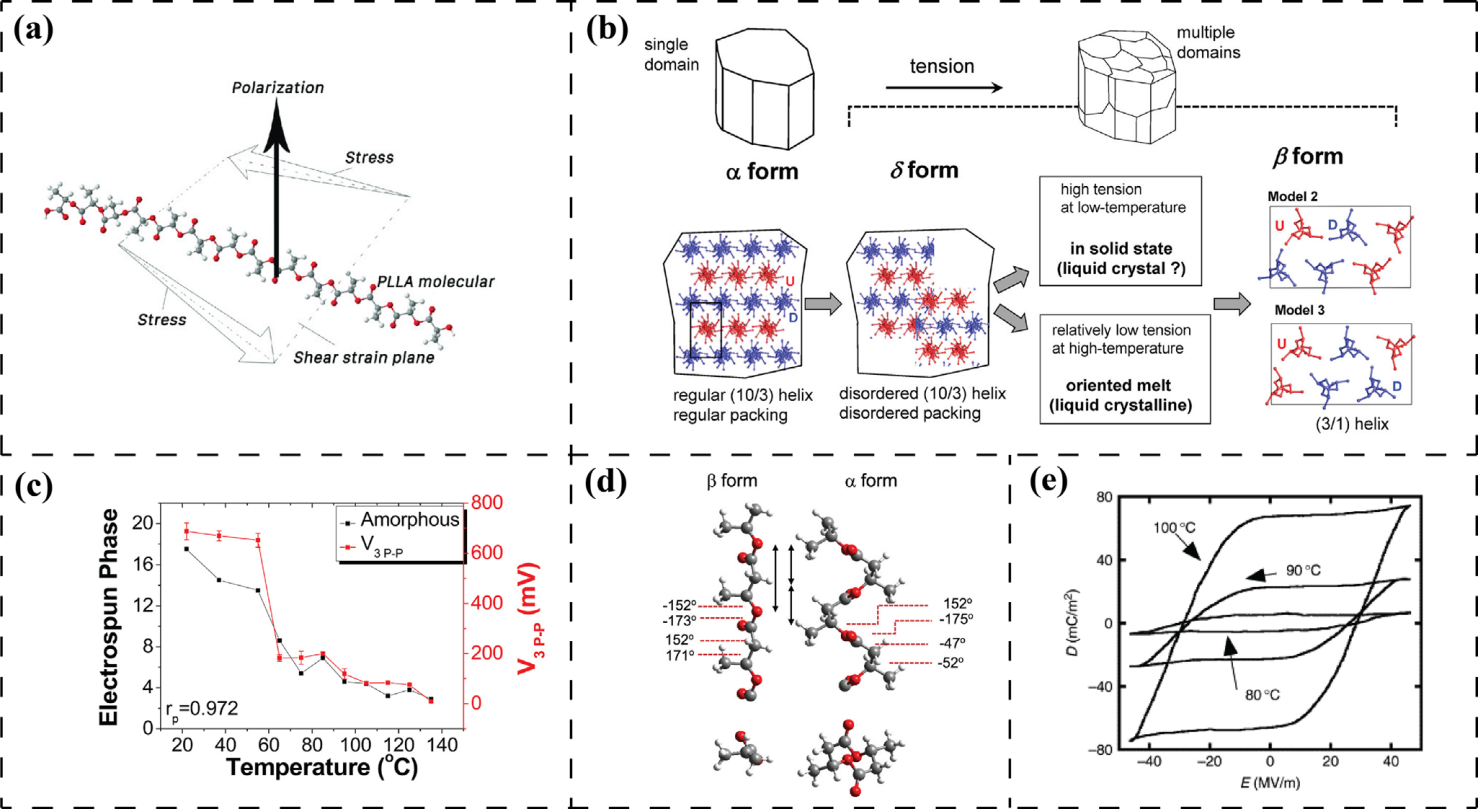
Piezoelectricity originating from [─(C═O)─] dipoles. Schematic representation of a) shear force‐induced polarization of a PLLA molecular chain. Reproduced with permission.^[^ ^88^ ^]^ Copyright 2020, WILEY‐VCH Verlag GmbH & Co. KGaA; b) phase transition in PLLA from *α*‐phase with orthorhombic packing of 10/3 helical chains in large intact domain to *α’* (*δ*)‐phase with disordered packing of 10/3 helical chains in smaller domains, and then *β*‐phase with trigonal packing of 3/1 helical chains in smaller domains, initiated by tension. Reproduced with permission.^[^ ^91^ ^]^ Copyright 2017, American Chemical Society. c) FTIR derived results for the amorphous content and longitudinal voltage output (*V*_3_) as a function of heat treatment temperature. Reproduced with permission.^[^ ^92^ ^]^ Copyright 2021, Elsevier. d) Molecular models of 2/1 helical chain conformation in PHB *α*‐phase and trans‐zigzag chain conformation in *β*‐phase. Reproduced with permission.^[^ ^93^ ^]^ Copyright 2019, American Chemical Society. e) Polarization displacement‐electric field hysteresis loops of PHB in a temperature range from 80 to 100 °C. Reproduced with permission.^[^ ^94^ ^]^ Copyright 2006, Elsevier.

PLLA is a biocompatible and biodegradable semi‐crystalline polyester exhibiting piezoelectric properties due to the molecular [─(C═O)─] dipoles bonded to the helical carbon skeleton.^[^ ^86^, ^87^ ^]^ A single PLLA chain undergoing shear stress on the plane where the helical centre axis is located results in the deformation and twisting of the carbon skeleton. Subsequently, the polar dipoles reorient themselves perpendicularly to the plane of the acting force, thus producing a net polarization fluctuation (**Figure** **4**a).^[^ ^88^, ^89^ ^]^ Therefore, the importance of the highly ordered crystalline regions of PLLA for its piezoelectricity can hardly be overstated. The discussion on the piezoelectric characterization of different crystalline conformations of PLLA has centered on mesophase, *α*’ (*δ*), *α*, and *β*. However, unlike PVDF, the molecular chains in PLLA, consistently follow a chiral helical backbone configuration TT'G, across all crystalline phases.^[^ ^90^ ^]^ This structural feature ensures that changes in PLLA's crystalline conformation do not yield a linear distribution of [─(C═O)─] dipoles, pointing perpendicularly to the same side of the molecular chain, as observed in the [─CF_2_─CH_2_─] dipoles of β‐phase PVDF. Instead, the different PLLA crystalline phases are distinguished by the helical pitch and spatial stacking of the parallel helical chains.^[^ ^91^ ^]^

The mesophase, *α’* (*δ*)‐phase, and *α*‐phase of PLLA typically follow the stacking of 10/3 helical chains in disordered small domains, intermediate domains with reduced disorder, and enlarged domains arranged in a fully ordered orthorhombic manner.^[^ ^95^, ^96^ ^]^ Ben Achour et al.^[^ ^97^ ^]^ demonstrated that both stress‐induced fully oriented mesophase and *α’*‐phase can contribute to the shear piezoelectricity of PLLA. They separated the contents of the mesophase and *α’*‐phase via analysing the wide‐angle X‐ray scattering (WAXS) data and compared them with the shear piezoelectric performance of PLLA films. The shear piezoelectric coefficients of fully oriented 100% PLLA mesophase and 100% PLLA *α’*‐phase were calculated to be 13 pC/N and 16 pC/N, respectively. Nevertheless, the acquisition of 100% single‐phase exists only in theoretical calculations. The reported optimum shear piezoelectric coefficient of PLLA reached 5.2 pC/N at 18% *α’*‐phase and 8% mesophase.^[^ ^97^ ^]^ For the orthorhombically packed 10/3 helical PLLA chains, i.e., PLLA *α*‐phase, the shear piezoelectric coefficient *d*_14_ of highly oriented films has been demonstrated to rise with increasing crystallinity, achieving 13.1 pC/N at 61% crystallinity.^[^ ^89^ ^]^ Additionally, the presence of the longitudinal piezoelectric response (around *d*_33_ = 1 pC/N) perpendicular to the *α*‐phase chain orientation has been reported, but its mechanism of electromechanical energy conversion at the molecular level remains unclear.^[^ ^88^, ^98^ ^]^

The transition of PLLA from *α*‐phase to *β*‐phase is associated with chain conformation and domain structures (Figure 4(b)).^[^ ^91^ ^]^ The slightly relaxed 3/1 helical pitch and trigonal chain stacking of PLLA *β*‐phase are usually achieved under stringent acquisition conditions, including high tension stretching below the melting temperature (*T*_m_),^[^ ^99^ ^]^ superheated stretching,^[^ ^91^ ^]^ and supercooled shear forces.^[^ ^100^ ^]^ When viewed perpendicular to the helical axis, the change in helical pitch may lead to a transition from a 360° distribution of [─(C═O)─] dipoles along the 10/3 helical chain (α‐phase) to a 120° spaced distribution on the 3/1 helical chain (β‐phase). The *β*‐phase PLLA has been reported to possess a high shear piezoelectric coefficient, *d*_14_ = 19 pC/N, achieved by modulating the crystallinity and domain orientation.^[^ ^101^ ^]^ However, the influence of the unique [‐(C = O)‐] dipole distribution and chain stacking mode on the piezoelectric properties requires further investigation.

The ferroelectricity of PLLA, particularly the behavior of [─(C═O)─] dipoles under a switching poling field, has been controversial. Typically, the lack of crystal symmetry is considered to result in a non‐ferroelectric property.^[^ ^102^ ^]^ However, Qing et al.^[^ ^103^ ^]^ have shown that PLLA thin films, crystaliszd directly from the melt state via annealing, can exhibit standard ferroelectric hysteresis loops within the high temperature range of 110—130 °C. As the testing temperature increases, PLLA exhibits a gradually decreasing *E*_c_ as well as a progressively increasing *P*_r._ Moreover, Jiang et al.^[^ ^104^ ^]^ demonstrated that a sufficiently strong electric field can cause the [─(C═O)─] dipoles to twist even at room temperature. However, the increase in crystallinity and the expansion of the crystals that accompany the rise in temperature can result in the disappearance of such twist. This finding resonates with the observations made by Tai et al. concerning the piezoelectric behavior of PLLA nanofibers.^[^ ^92^ ^]^ They found that PLLA nanofibers (diameter *D* ranging from 15 to 525 nm), fabricated through electrospinning, exhibited a *D*‐dependent longitudinal piezoelectric coefficient prior to heat treatment. Nanofibers with *D* = 15 nm exhibited *d*_33_ ≈ 27.5 pm V^−1^, which dropped exponentially to *d*_33_ ≈ 5 pm V^−1^ as *D* increased to 100 nm or beyond. However, when all nanofibers underwent annealing treatment above the glass transition temperature (*T*_g_), the longitudinal piezoelectric response showed a dramatic decrease, while the shear piezoelectric response appeared to increase. The longitudinal piezoelectric response was observed to correlate with the changes in amorphous phase content, while the shear piezoelectric response exhibited a strong correlation with the *α*’ phase content (Figure 4c). They hypothesized that above the *T*_g_, the crystallisation process of PLLA could reorient the molecular chains, thus disrupting the alignment of the [─(C═O)─] dipoles that were induced by the high voltage field and the drawing forces exerted during electrospinning.

In addition to PLLA, the [─(C═O)─] dipole is also present in PHB, a biodegradable polymer, where the *β*‐phase is primarily responsible for its piezoelectric properties. The left‐handed 2/1 helical PHB chains (TTGG) are arranged in an orthorhombic configuration, which characterizes the *α*‐phase.^[^ ^93^ ^]^ Stress‐induced *β*‐phase transformation involves the complete extension of PHB helical chains, resulting in the hexagonal packing of trans‐zigzag (TTT) chains (Figure 4d). Notably, the unique trans‐zigzag chain conformation of the PHB *β*‐phase leads to an isotropic arrangement of [─(C═O)─] dipoles. Although the mechanism by which the PHB *β*‐phase responds to external stress remains unclear, the formation of highly oriented as well as highly crystalline *β*‐phase has been demonstrated to drive the longitudinal and transverse piezoelectric coefficients of PHB to an astonishing *d*_33_ = 26.8 pC N^−1^ and 25.7 pC N^−1^, respectively.^[^ ^105^ ^]^ In addition, the free dipoles in the untreated low crystallinity state of PHB exhibit ferroelectric switching at 80–100 °C (Figure 4e).^[^ ^94^ ^]^ However, the highly aligned crystals that form within the PHB matrix after annealing have been demonstrated to result in the disappearance of the polarization displacement‐electric field hysteresis curve.

#### 2.2.3 [─NH─C(═O)─] Dipole

**Figure 5:**
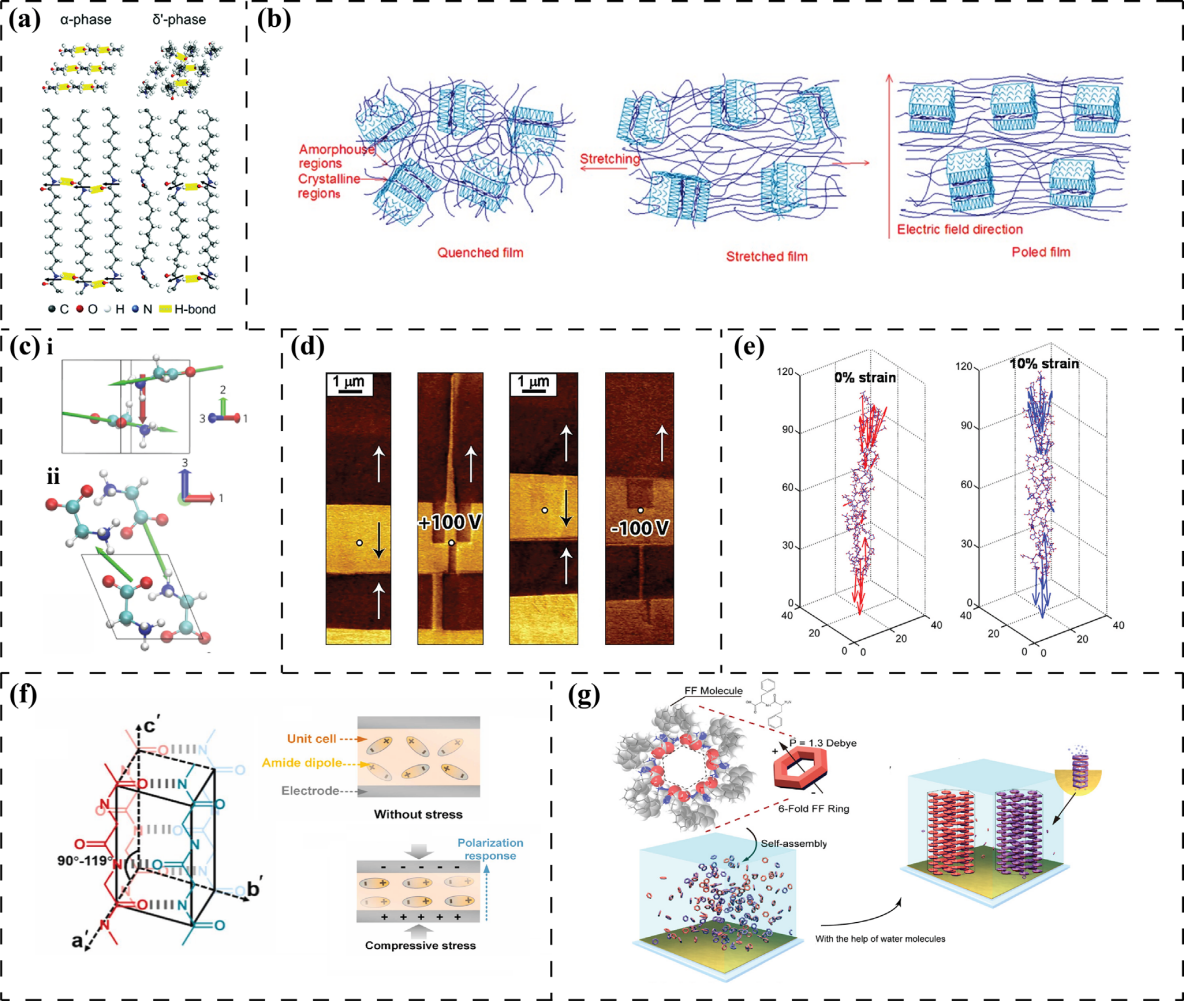
Piezoelectricity originating from [─NH─C(═O)─] dipoles. a) Molecular models of PA 11 *α*‐phase and *γ* (*δ’*)‐phase. Reproduced under the terms of the CC BY 3.0 Licence.^[^ ^110^ ^]^ Copyright 2018, Royal Society of Chemistry. b) Schematic illustration of stretching‐ and electrical poling‐induced reorientation of *γ*‐crystals in PA 11. Reproduced with permission.^[^ ^114^ ^]^ Copyright 2014 Wiley Periodicals. (c_i_) Molecular dipoles in *β*‐glycine (green arrows) sum to the spontaneous dipole moment (red arrow) in the crystal form of *β*‐glycine; (c_ii_) net polarization (green arrow) generated along 1‐axis when deformed in shear plane normal to three‐axis. Reproduced with permission.^[^ ^119^ ^]^ Copyright 2017, Springer Nature. d) Local polarization reversal in domain structures of *β*‐glycine crystals. Reproduced under the terms of the CC BY 4.0 Licence.^[^ ^120^ ^]^ Copyright 2019, MDPI. e) Change of dipole moment vectors of all residues in the collagen molecule before (red arrows) and after (blue arrows) 10% strain. Reproduced with permission.^[^ ^121^ ^]^ Copyright 2016, American Chemical Society. Schematic representation of (f) SF *β*‐sheet conformation and the reorientation of [─NH‐C(= O)‐] dipoles under compression. Reproduced with permission.^[^ ^122^ ^]^ Copyright 2024, Elsevier. (g) Self‐assembly of 6‐FF rings. Reproduced with permission.^[^ ^123^ ^]^ Copyright 2023, American Chemical Society.

The polar amide group [─NH─C(═O)─] (Figure 2e) gives rise to a net molecular dipole moment of 3.7 Debye due to the difference in electronegativity of O═C and N─H bonds.^[^ ^106^, ^107^ ^]^ The synthetic semi‐crystalline polyamide (PA) family [─NH─(CH_2_)─C(═O)─]_n_ is a notable example and is considered a promising candidate for BTE applications due to its high elastic modulus, resistance to microbial contamination, and biocompatibility.^[^ ^108^, ^109^ ^]^ Each amide group is comprised of oppositely arranged N─H and C═O bonds. The number of carbon atoms in each repeating segment determines whether a PA is classified as odd or even numbered. The odd‐numbered PAs (i.e., PA 11 or PA 5) are considered as the subcategory exhibiting a piezoelectric response. This characteristic arises from the presence of methylene group [─(CH_2_)─] in the repeating units, which share a similar planar zigzag conformation to that of the PVDF chain. The odd‐numbered segments ensure a consistent directionality of the net dipole moment arising from the polar amide bonds [─NH─C(═O)─] along a single PA molecular chain (**Figure** **5**a).^[^ ^110^, ^111^ ^]^ Compared to the net dipole moment in the PVDF monomer, the single amide group [─NH─C(═O)─] exhibits a higher net dipole moment. However, due to the presence of the methylene segments, the spatial density of net dipole moments on PA chains is “diluted”. Herein, the reduced number of repeated,[─(CH_2_)_x_─] i.e., from PA 11 to PA 5, leads to the increased dipole density from 1.34 $\mathrm{Debye}/100{\dot{A}}_{3}$ to 2.94 $\mathrm{Debye}/100{\dot{A}}_{3}$, respectively.^[^ ^112^ ^]^

During PA crystallization, different conditions lead to the variation in the spatial distribution of intermolecular hydrogen bonds that stack the adjacent molecular chains, thus leading to varied crystal conformations (Figure 5a).^[^ ^110^ ^]^ Within the PA 11 *α*‐phase produced from slow crystallization from the melt, all of the [─NH─C(═O)─] dipoles are anti‐parallel and tightly hydrogen‐bonded, forming a thermodynamically stable triclinic packing lattice conformation.^[^ ^113^ ^]^ Although the *α*‐phase has a crystallographic arrangement to facilitate excellent mechanical properties, it has a low piezoelectric constant (*d*_33_ < 1 pC/N).^[^ ^110^, ^114^ ^]^ Conversely, if treated by high supercooling, PA 11 crystals follow the sparsely‐packed non‐centrosymmetric pseudo‐hexagonal *γ* or *δ’* conformation.^[^ ^115^ ^]^ The intermolecular hydrogen bonds endow a less restricted reorientation of [─NH─C(═O)─] dipoles. Subsequently, upon the application of mechanical drawing or electrical field poling (Figure 5b), a higher piezoelectric constant (*d*_33_ = −3.9 pC N^−1^) as well as ferroelectricity is observed.^[^ ^110^, ^114^, ^116^ ^]^ Besides, similar arrangements of [─NH─C(═O)─] dipoles can be found in odd‐numbered aliphatic polyurea (i.e., polyurea 5), which has been reported to exhibit significant piezoelectricity (*d*_31_ = 4 pC N^−1^).^[^ ^117^ ^]^ Crystallization of polyurea, via rapid quenching followed by recrystallization above 120 °C, enables production of a stacked lamellar structure with compactly folded molecular chains.^[^ ^118^ ^]^ If future studies can elucidate that the folding dynamics within these lamellar crystals are conducive to the unidirectional arrangement of polar groups, the potential of polyurea in piezoelectric applications will be enhanced.

The [─NH─C(═O)─] linkage is commonly referred to as a peptide bond in natural polymers (e.g., collagen), that forms through a dehydration reaction, i.e., the elimination of a water molecule (H_2_O) between a carboxyl group (─COOH) and an amino group (─NH_2_) of the linked amino acids in proteins. Guerin et al.^[^ ^119^ ^]^ performed density functional theory (DFT) calculations on individual amino acids, including *β*‐glycine, and demonstrated that the dipole moment points from the more electronegative carboxyl groups (─COOH) toward the amino groups (─NH_2_). This contributes to a spontaneous dipole moment in the crystalline form of *β*‐glycine (Figure 5c_i_). When deformed in the shear plane normal to the three‐axis, the component of the generated strong net polarization in one‐axis refers to the shear piezoelectricity of *β*‐glycine crystals (Figure 5c_ii_). The coefficient was predicted to be *d*_16_ = 195 pm V^−1^, that aligns with the experimental value of 178 pm V^−1^.^[^ ^119^ ^]^ A similar method was used to explore the piezoelectric manifestation of multiaxial juxtaposed amino acid crystals.^[^ ^124^ ^]^ The piezoelectric charge tensors of various amino acid residues, including L‐proline, hydroxy‐L‐proline, and L‐alanine in their crystalline forms, have demonstrated a shear piezoelectric response (coefficients ranging from 0.09 to 27.75 pC N^−1^ in absolute value). Additionally, *β*‐glycine crystals have been shown to exhibit polarization reversal when exposed to a PFM probe charged with a pulse voltage (*U* = ±100 V), thus demonstrating their ferroelectricity (Figure 5d).^[^ ^120^ ^]^ It is worth noting that PFM is a widely used technique for probing piezoelectric and ferroelectric properties, especially in nanostructured and thin‐film biomaterials. In short, by applying a voltage to the PFM tip, a localised polarization field is generated at the tip‐sample interface.^[^ ^125^ ^]^ When the applied field is below the coercive field, the resulting surface deformation enables quantitative estimation of the piezoelectric coefficients. When the applied field exceeds the coercive field, if present, the ferroelectric domains switch as a result of the reorientation of dipoles, which is typically accompanied by the formation of a piezoresponse hysteresis loop.^[^ ^125^ ^]^

Higher‐dimensional structures utilizing amino acids as building blocks, i.e., polypeptides and proteins, differ in their specific amino acid sequence and subsequent structural conformation. Type I collagen, the most abundant protein in vertebrates and widely used in BTE applications, possesses a distinctive molecular structure that imparts notable piezoelectric properties. The triple *α*‐helical conformation of the parallel sequences of [‐glycine‐X‐Y‐]_n_ is constituted by alternately changing X and Y, i.e., glycine, proline, and hydroxyproline, and is stabilized by intrachain hydrogen bonds.^[^ ^126^ ^]^ Unlike the planar zigzag arrangement of molecular dipoles in the δ′‐phase PA 11, the tropohelical packing of the [‐glycine‐X‐Y‐]_n_ chains endows collagen fibrils with a structurally complex and non‐centrosymmetric nature. Molecular dynamics (MD) simulations conducted by Ravi et al.^[^ ^127^ ^]^ indicate that the polar [─NH─C(═O)─] dipoles along the glycine‐proline/hydroxyproline backbone undergo reorientation when the collagen fibril is subjected to either compression or tension in the fibril length direction, as the result of winding or unwinding, respectively. Such dipole reorientation induces fluctuations in the net dipole moment, thereby affecting the polarization along the entire collagen fibril, as shown in Figure 5e.^[^ ^121^, ^127^ ^]^ Furthermore, an atomistic simulation model developed by Zhou et al. revealed that, along with the inclination of [─NH─C(═O)─] dipoles, variations in their magnitude under stress also play an equally crucial role in the overall polarization behavior of the collagen fibril.^[^ ^121^ ^]^ Based on DFT calculations, Guerin et al.^[^ ^124^ ^]^ predicted the longitudinal piezoelectric constant for collagen fibril (*d*_33_ = 0.88 pC N^−1^), that is closely aligned with the experimental value (*d*_33_ = 0.89 pC N^−1^) from PFM measurement.^[^ ^128^ ^]^ Nevertheless, the complex spatial arrangement of amino acid residues along the collagen chains does not favor the development of their overall ferroelectricity. No polarization switching can be observed during the PFM measurements of the collagen fibril.^[^ ^129^, ^130^ ^]^ Alternative amino acid based *α*‐helical structures have been explored for their piezoelectric properties, including keratin (*d*_33_ up to −1.8 pC N^−1^),^[^ ^131^, ^132^ ^]^ and poly(γ‐benzyl‐l‐glutamate) (PBLG) (*d*_33_ up to 25 pC N^−1^).^[^ ^133^ ^]^ Using amino acids as building blocks, the design and organization of [─NH─C(═O)─] dipoles via supramolecular chemistry offer promising candidates for applications in BTE. For further details, readers are encouraged to refer to the comprehensive review by Tayi et al.^[^ ^134^ ^]^

Compared to the *α*‐helical structure, the net dipole moment of the *β*‐sheet structure is usually considered to be modest, as the alternating distribution of amino acid residues on both sides of the chain cancel themselves out, leaving only a weak net dipole moment along the chain direction.^[^ ^135^, ^136^ ^]^ However, silk fibroin (SF), a semi‐crystalline biomaterial containing a *β*‐sheet microstructure, shows piezoelectric properties and has gained attention for BTE due to its outstanding mechanical properties, biocompatibility, biodegradability, surface modifiability, and osteogenic induction.^[^ ^135^, ^137^, ^138^ ^]^ When the monoclinic cells of stacked antiparallel SF chains in the *β*‐sheet are subjected to an external force, the rotation of [─NH─C(═O)─] dipoles accompanying the intra‐ and interchain movement leads to electrical polarization and a piezoelectric response (Figure 5f).^[^ ^122^ ^]^ Subsequently, SF has noticeable piezoelectric properties, including energy conversion efficiency (up to 66%),^[^ ^139^ ^]^ shear piezoelectric constant (*d*_14_ up to 1.5 pC N^−1^),^[^ ^140^ ^]^ longitudinal piezoelectric constant (*d*_33_ up to 7.7 pm V^−1^),^[^ ^122^ ^]^ and response speed (as short as 3.4 ms).^[^ ^141^ ^]^ Moreover, Sencadas et al.^[^ ^142^ ^]^ evaluated the ferroelectric behavior of SF nanofibrils using PFM. The amplitude and phase profile of the SF nanofibrils, as a function of the applied switching electric field, suggest the reversibility of the dipoles, which is a specific characteristic of ferroelectric materials.

Additionally, the unique [─NH─C(═O)─] dipole arrangement is demonstrated in the laboratory‐synthesised self‐assembled short peptide motif, diphenylalanine (FF).^[^ ^143^ ^]^ The six FF peptides form a ring structure joined by NH · · · O hydrogen bonds.^[^ ^144^ ^]^ The unique ring assembly allows the dipole moments of each peptide bond to be oriented along the same direction, perpendicular to the ring plane, thus resulting in a net dipole moment equivalent to 1.3 Debye (Figure 5g).^[^ ^123^, ^144^ ^]^ The 6‐FF ring exhibits spontaneous polarity and is attracted to the negatively charged basal electrode. Thereby, the attached 6‐FF ring functions as a nucleus that initiates the isotropic stacking of free 6‐FF rings suspended in the solution, and eventually grows into an FF nanotube.^[^ ^145^ ^]^ FF nanotubes obtained by dip‐coating and slow pulling exhibited near‐perfect unidirectional alignment as well as an excellent shear piezoelectric constant *d*_15_ = 46.6 pm V^−1^.^[^ ^145^ ^]^ Moreover, Su et al.^[^ ^123^ ^]^ achieved a uniform polarization of FF microrods on a silver‐coated substrate by applying an evenly distributed high voltage electric field between the substrate and the parallel electrodes during the FF microrod growth. A remarkably high longitudinal piezoelectric constant *d*_33_ = 18.91 pm V^−1^ was produced. These studies indicate that the self‐assembly properties of FF ensure reproducible and excellent piezoelectric properties even with simple preparation methods. Furthermore, FF nanotubes are highly biocompatible peptides with remarkable mechanical properties in their derived structures, making them promising biomaterials for developing piezoelectric‐functionalized bone repair scaffolds.^[^ ^146^, ^147^ ^]^

#### 2.2.4 OH · · · O Dipole

As previously discussed, hydrogen bonding (Figure 2f) plays a crucial role in the structure of polypeptide/protein molecular chains as they arrange into a favorable conformation. However, hydrogen bonds, being relatively weak non‐covalent bonds, are normally overlooked for their potential to function as dipoles.^[^ ^148^ ^]^ Sohn et al.^[^ ^149^ ^]^ have exploited a high‐voltage poling field to disrupt the hydrogen bonds of SF nanofibrils. They indicated that the disruption of hydrogen bonds can lead to a significant decrease in *P*_r_ and the piezoelectric coefficient of SF nanofibrils. This demonstrates the contribution of hydrogen bonds as dipoles. In addition, Ghosh et al.,^[^ ^150^ ^]^ using PFM, identified a hysteresis response in the amplitude of gelatin nanofibers (GNF) under an alternating high voltage. This was attributed to the reorientation of the hydrogen (OH · · · O) dipoles formed between peptide chains. From the perspective of the molecular structure, it appears that the hydrogen bonding network and the ordered spatial stacking of peptide chains are inextricably linked. Disentangling the contribution of [─NH─C(═O)─] dipoles and OH · · · O dipoles to the overall piezoelectricity presents a significant challenge.

**Figure 6:**
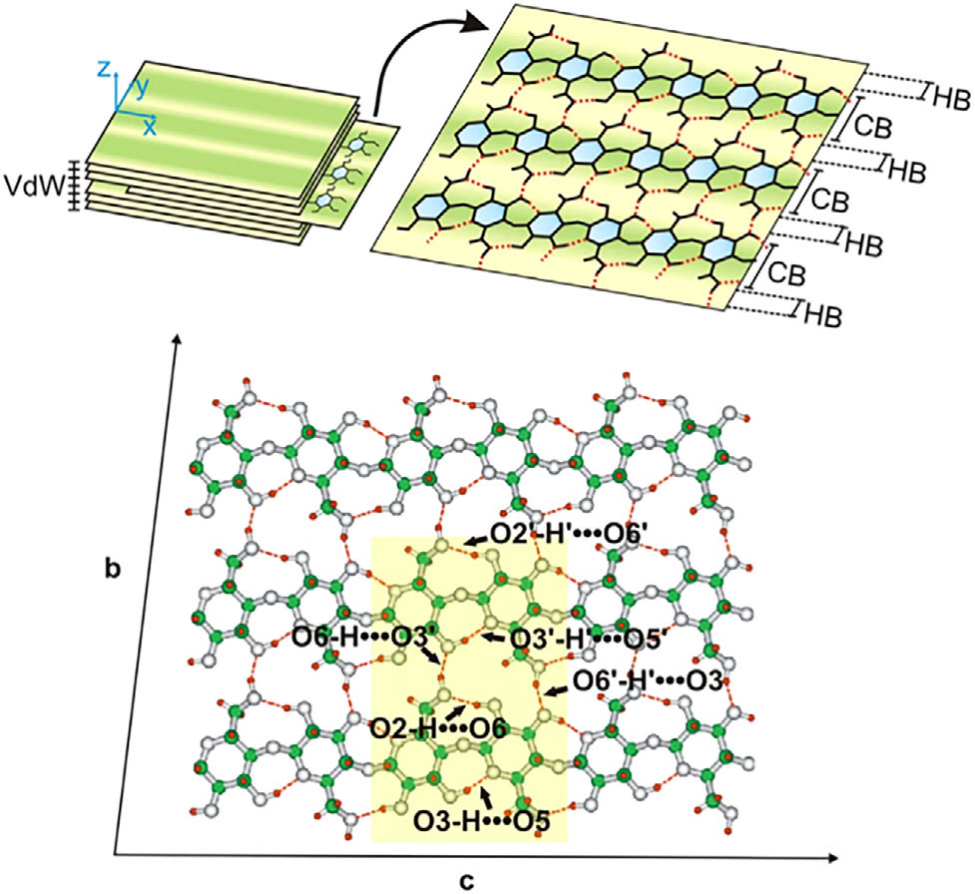
Schematic illustration of molecular chain conformation and hydrogen bonding networks in 2D cellulose I_β_ crystals. Reproduced under the terms of the CC BY 4.0 Licence.^[^ ^152^ ^]^ Copyright 2016, Springer Nature.

The piezoelectric response of cellulose, a polysaccharide rich in hydrogen bonds, is receiving increasing interest.^[^ ^151^ ^]^ With the more sensitive non‐local DFT method, Garcia et al.^[^ ^152^ ^]^ for the first time, implemented the coupling of the electric field and strain tensor in the bond region for the intermolecular O6‐H · · · O3’, intramolecular O3‐H · · · O5, and intramolecular O2‐H · · · O6 hydrogen bonds within 2D cellulose I_β_ crystals (**Figure** **6**). The piezoelectric coefficients of these OH · · · O dipoles were computed to be 4.3, 10.0, and 36.4 pm V^−1^, respectively. Direct piezoelectric measurement of randomly oriented I_β_ cellulose nanofibers (CNFs) revealed that the longitudinal piezoelectric coefficient reached *d*_33_ = 4.7 pC N^−1^.^[^ ^153^ ^]^ Moreover, in the ferroelectric hysteresis evaluation, the I_β_ CNFs did not exhibit hysteresis until the electric field strength was elevated to 40 MVm^−1^. Nevertheless, even when the field strength was further increased to 50 MV m^−1^, the P_r_ of I_β_ CNFs was still as low as 0.15 µC cm^−2^. Further acidic hydrolysis of the amorphous region of CNFs yields cellulose nanocrystals (CNC) with smaller dimensions and higher crystallinity,^[^ ^154^ ^]^ that impart higher piezoelectric properties (*d*_33_ = 4.7 pC N^−1^).^[^ ^155^ ^]^

From a molecular structure perspective, the various arrangements of hydrogen bonding result in the conformational differences observed in the cellulose crystalline allomorphs, i.e., I_β_, I_α_ and II.^[^ ^156^ ^]^ The cellulose I_α_ crystal can be obtained from bacteria and alga.^[^ ^157^, ^158^ ^]^ Cellulose II is typically regenerated from dissolved native cellulose, with inter‐and intramolecular hydrogen bonds disrupted by solvents.^[^ ^159^ ^]^ Both cellulose I_α_ and II have been proved to possess non‐negligible potential for piezoelectric applications. For instance, a piezoelectric sensor produced from cultured wild‐type *K. xylinus* strains exhibited outstanding crystallinity (≈92%) and longitudinal piezoelectric coefficient (*d*_33_ = 16.8 pC N^−1^).^[^ ^160^ ^]^ Besides, the regenerated cellulose II film has been demonstrated to possess remarkable transverse piezoelectric coefficient *d*_31_ up to 27.3 pC N^−1^.^[^ ^161^ ^]^ Similarly, progress has been made in evaluating the piezoelectricity of other polysaccharides, e.g., chitin (0.1–0.3 pm V^−1^)^[^ ^162^ ^]^ and chitosan (*d*_33_ up to 18.6 pC N^−1^).^[^ ^163^ ^]^ Although investigations to elucidate the mechanism of the piezoelectric response at the molecular level of these polysaccharides remain scarce, the electromechanical conversion of OH · · · O dipoles could serve as a potential entry point.

## 3 Pseudo‐Piezoelectricity of Non‐Polar Materials

### 3.1 Charged Voids – Piezoelectrets

For piezoelectric materials, polarity seems to be an indispensable prerequisite. However, nonpolar materials can possess piezoelectric properties and even exceed those of conventional piezoelectric materials by creating highly aligned charged microcavities/voids inside the matrix.^[^ ^61^, ^164^ ^]^ The gas trapped in these voids, formed during material preparation, is polarized by applying a high electric field via contact/corona poling. When the applied electric field exceeds the Paschen breakdown threshold of the gas, dielectric barrier discharges may occur. Opposite charges are captured by the opposing void walls along the radial direction of the polarized electric field, thereby generating a macroscopic dipole moment inside each void (Figure 2g).^[^ ^61^, ^164^ ^]^ Such composition of polymer and charged voids is termed as piezoelectrets.

A classic example is the non‐piezoelectric polypropylene (PP) combined with air, that shows an impressive longitudinal piezoelectric coefficient *d*_33_ (208 pC/N at 20 °C) after thermal treatments and corona poling.^[^ ^165^ ^]^ Similar results have been reported using polytetrafluoroethylene (PTFE) foams (*d*_33_ up to 2700 pC N^−1^),^[^ ^166^ ^]^ polyurethane (PU) foams (*d*_33_ up to 244 pC N^−1^),^[^ ^167^ ^]^ and PLA foams (*d*_33_ and *d*_31_ up to 596 and 44 pCN^−1^).^[^ ^168^, ^169^ ^]^ As a trade‐off for an ultra‐high electromechanical sensitivity, the mechanical strength of these piezoelectrets is low. Moreover, the piezoelectric properties are significantly affected by temperature and time, i.e., a significant drop in piezoelectric coefficients with increasing temperature and extended storage time.^[^ ^166^, ^167^, ^168^, ^169^ ^]^ Subsequently, considering BTE applications, although PP piezoelectrets are regarded as biocompatible and non‐cytotoxic to osteoblasts, it remains unclear whether these piezoelectrets can function properly in physiological environments over the long‐term.^[^ ^170^ ^]^

### 3.2 Diffusing Ions – Piezoionics

From the superficial characteristics of piezoelectric materials, piezoelectricity can also be interpreted as a strain‐generated potential (SGP), that can be observed in bone and cartilage and is not solely associated with collagen.^[^ ^20^, ^32^ ^]^ Poillot et al.^[^ ^171^ ^]^ commented that in addition to the role of piezoelectric biomolecules, the streaming potential dominated by ions and fluids also contributes to the generation of an SGP. The external compressive strain on the extracellular matrix (ECM) leads to a denser packing of macromolecules with fixed surface charges compared to the neighbouring ECM. Thus, a higher fixed charge density is formed, and a charge gradient between the compressed ECM and the adjacent region is established. This gradient drives ionic diffusion.^[^ ^171^ ^]^

This bio‐inspired mode of strain‐ionic diffusion facilitates the emergence of the nascent concept of piezoionics.^[^ ^172^ ^]^ For example, Liu et al.^[^ ^173^ ^]^ prepared piezoionic membranes using non‐piezoelectric thermoplastic polyurethane as the polymer matrix, ionic liquid 1‐ethyl‐3‐methylimidazolium bis(trifluoromethylsulfonyl) imide as the source of free ions, and graphene oxide as the electrodes, and successfully demonstrated their potential in motion capturing sensors. Additionally, using piezoionic films made of non‐piezoelectric polyacrylamide and sodium chloride, Dobashi et al.^[^ ^62^ ^]^ revealed the possibility of self‐powered current pulses to stimulate the sciatic nerve in rodents (Figure 2h). However, it is currently unrealistic to over‐interpret the potential of piezoionics for bone repair applications due to the limited data. Further studies are required to understand the underlying mechanism of piezoionics as well as their applicability within biological environments.

## 4 Engineering the Dipoles During Fabrication

The emphasis of piezoelectricity in BTE is evolving from the initial phase, which investigates the presence or absence of a piezoelectric response in osteogenesis, to a more advanced phase that focuses on steering piezoelectric parameters to guide bone regeneration at the cellular level, i.e., cell adhesion, proliferation, osteogenic differentiation, cell maturation, and biomineralization. The key to success is associated with the capability of modulating the mode, amplitude, and efficiency of piezoelectricity. Furthermore, controlling the dipoles or asymmetric center of the material matrix is essential. This section categorizes current engineering methods that directly or indirectly modulate the dipoles or asymmetric center during fabrication, rather than the fabrication methods of piezoelectric scaffolds that have been detailed in several relevant reviews.^[^ ^36^, ^174^ ^]^

### 4.1 Electrical Modulation

**Figure 7:**
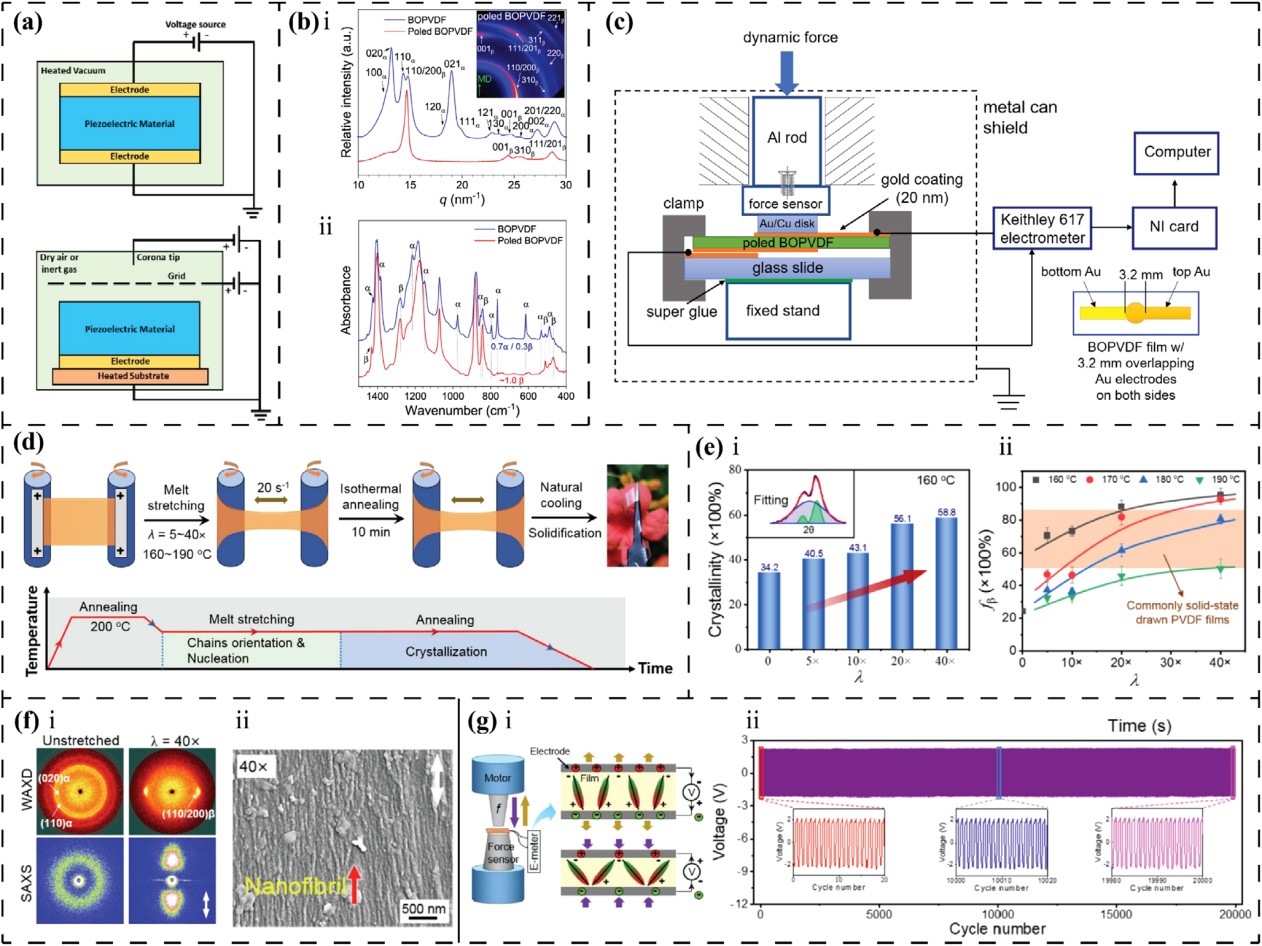
Electrical modulation of dipoles. a) Schematics of contact DC poling and non‐contact corona poling. Reproduced under the terms of the CC BY 4.0 Licence.^[^ ^175^ ^]^ Copyright 2021, Wiley‐VCH GmbH. Comparison between b_i_) Wide angle X‐ray diffraction (WAXD) analysis and b_ii_) FTIR spectrum of non‐poled BOPVDF and poled BOPVDF. Reproduced under the terms of the CC BY 4.0 Licence.^[^ ^177^ ^]^ Copyright 2021, Springer Nature. c) Schematic of the experimental setup for *d*_33_ measurement. Reproduced under the terms of the CC BY 4.0 Licence.^[^ ^177^ ^]^ Copyright 2021, Springer Nature. Tensile modulation of dipoles. d) Schematic of multi‐step melt drawing of PVDF films. Reproduced with permission.^[^ ^178^ ^]^ Copyright 2022, Elsevier. Dependence of e_i_) crystallinity on *D*_r_ and e_ii_) *F_β_* on *T* and *D*_r._ Reproduced with permission.^[^ ^178^ ^]^ Copyright 2022, Elsevier. f_i_) 2D WAXD and small‐angle X‐ray scattering (SAXS) analysis of unstretched film and PVDF film stretched with optimum conditions; f_ii_) SEM observation of highly aligned nanofibrils on the surface of PVDF film stretched with optimum conditions. Reproduced with permission.^[^ ^178^ ^]^ Copyright 2022, Elsevier. g_i_) Schematic of piezoelectric measurement; g_ii_) piezoelectric performance of PVDF device stretched with optimum conditions for long‐term stability. Reproduced with permission.^[^ ^178^ ^]^ Copyright 2022, Elsevier.

High voltage electrical poling is a common method used to induce the desired alignment of ferroelectric dipoles/domains within either organic or inorganic piezoelectric materials. Electrical poling is usually divided into two types: contact direct current (DC) poling and non‐contact corona tip poling (**Figure** **7**a).^[^ ^175^ ^]^ Contact DC poling involves the use of two parallel charged electrodes that connect opposing surfaces of a ferroelectric material. This technique is widely used due to its simple experimental setup and intuitive parameter adjustment. However, the increased voltage of the applied electric field is accompanied by a significant increase in the risk of poling failure due to electrical breakdown. Once a breakdown path appears between the two electrodes on opposing surfaces of the ferroelectric material, which is particularly likely to occur when poling porous constructs, all the charge may escape.^[^ ^176^ ^]^ Alternatively, non‐contact corona poling is conducted by creating a corona discharge between a high‐voltage tip and the surface of a ferroelectric material, with the opposing surface grounded. The poling field is formed between the grounded surface and the corona‐charged surface of the ferroelectric material. The presence of electrical breakdown paths does not result in the escape of all charges from the corona‐charged surface, thus maintaining a stable and high poling field during the poling process.^[^ ^176^ ^]^

The applicability of electrical poling to piezoelectric biomaterials substantially depends on the capability for ferroelectric switching of the dipoles/domains within a particular molecular/crystal structure, i.e., whether the piezoelectric biomaterials exhibit ferroelectricity. For inorganic ferroelectric biomaterials such as BT and KNN, high voltage poling is typically an inevitable process used to reorient ferroelectric domains that tend to align themselves along a series of crystallographic directions with the lowest free energy.^[^ ^55^, ^179^ ^]^ The orientation of the ferroelectric domains, as well as the piezoelectric properties after poling, strongly depend on the poling conditions, including poling field strength, poling temperature, and duration.^[^ ^180^, ^181^, ^182^, ^183^, ^184^ ^]^

In terms of organic ferroelectric biomaterials, electrical poling has been employed as an effective tool to modulate dipoles as well as their piezoelectricity. For instance, by applying repeated (40 times) contact DC poling (650 MV m^−1^) to fresh biaxially oriented PVDF (BOPVDF) membranes, Huang et al.^[^ ^177^ ^]^ observed that the poled BOPVDF exhibited a significantly higher *P*_r_, which was more than two times that of the unpoled BOPVDF. Moreover, wide‐angle X‐ray diffraction (WAXD) and FTIR spectroscopy experiments revealed the disappearance of all peaks assigned to the *α*‐phase, indicating the complete formation of a *β*‐phase crystal microstructure (crystallinity of ≈0.52) (Figure 7b). Therefore, the poled PVDF exhibited a high direct piezoelectric coefficient *d*_33_ = −62 pC/N, measured with the setup shown in Figure 7c. These findings illustrate that the electrical poling field not only modulates the alignment of [‐CF_2_‐CH_2_‐] dipoles, but also enhances the fraction of ordered ferroelectric phases.

However, the effectiveness of electrical poling for molecular dipoles is not universal. For instance, the modulation of electrical poling for [─(C═O)─] dipoles is restricted. Under the high voltage poling field, the reorientation of [─(C═O)─] dipoles within either PLLA or PHB occurs only in a specific poling temperature window.^[^ ^94^, ^103^ ^]^ Additionally, the availability of reorientable [─(C═O)─] dipoles decreases with increasing crystallinity of the material matrix. For [─NH─C(═O)─] dipoles, the utility of the poling field is manifested only when their spatial arrangement in molecular structure exhibits a clear preference, namely *γ*‐phase PA 11,^[^ ^114^ ^]^ *β*‐glycine crystals,^[^ ^120^ ^]^ and polar FF rings.^[^ ^123^ ^]^ Besides, the response of OH · · · O dipoles in the presence of poling fields has not been determined clearly. The ferroelectric hysteresis of I_β_ cellulose might appear only when the electric field rises to 40 MV m^−1^.^[^ ^153^ ^]^ Conversely, the high voltage poling process can cause the decomposition of the hydrogen bonding network as well as the “inverse depolarization” of SF.^[^ ^149^ ^]^

Additionally, in most cases of 3D printed porous piezoelectric scaffolds, the fabrication processes themselves, including selective laser sintering (SLS) and piezoelectric coating of 3D porous metallic scaffolds, do not involve the modulation of dipole moments.^[^ ^185^, ^186^, ^187^, ^188^, ^189^ ^]^ Therefore, electrical poling is necessary to impart piezoelectricity to these scaffolds. However, the electrical modulation of dipoles in 3D porous piezoelectric structures is challenging. Zhang et al.^[^ ^190^ ^]^ indicated that during the poling of porous piezoelectric constructs, the electric field tends to be concentrated in the air due to it having a lower dielectric constant. Alternatively, the electric field distribution within the solid piezoelectric material phase is lower. In their ferroelectric hysteresis measurements with controlled field strength, the increase in porosity led to a rising coercive field and a diminished *P*_r_, which significantly reduced the effectiveness of poling on the overall constructs.

In general, the electrically regulated reorientation of dipoles can be observed through variations in the piezoelectric coefficients. This correlates with a shift in the magnitude of the surface charge and surface potential (*Φ*) of the piezoelectric materials under stimulation. The oriented dipoles contribute to the macroscopic polarity of the piezoelectric material, which manifests as a resting *Φ* in the absence of stimulation. Moreover, the degree of dipole orientation determines the magnitude of *Φ*. Tang et al.^[^ ^28^ ^]^ employed scanning Kelvin probe microscopy to investigate the *Φ* of PVDF‐TrFE films poled under different contact poling fields (from 0 to ≈90 MV m^−1^). As the intensity of the poling field increased, the randomly oriented [─CF_2_─CH_2_─] dipoles gradually reoriented themselves along the direction of the poling field, thus leading to the enhancement of *Φ* from −3 to 915 mV (Figure 7c).

Depending on the sign of the voltage source applied to the ungrounded electrode during poling, as well as the direction of the applied poling field, the potentials of the opposing surfaces of the poled materials may exhibit varying signs and magnitudes. Considering the liquid environments, particularly in tissue engineering applications, the zeta potential (*Φ*_zeta_) at the slipping plane is commonly used to describe the interactions between biological factors and the material surface.^[^ ^191^ ^]^ The degree of dipole orientation remains simple for the modulation of the resting *Φ*_zeta_. Zhang et al.^[^ ^192^ ^]^ reported that increasing the temperature (*T*) during the electrical poling of PVDF‐TrFE films facilitated an increase in the fraction of oriented *β*‐phase, thus enhancing the magnitude of *Φ*_zeta_ and *d*_33_.

### 4.2 Thermal and Tensile Modulation

Thermal and tensile treatments are inextricably linked as methods for dipole modulation in piezoelectric biomaterials, especially for organic materials. Appropriate heat treatment can enhance crystallinity and facilitate the formation of a desired crystalline phase conducive to bulk piezoelectricity, e.g., *β*‐phase PVDF.^[^ ^193^ ^]^ However, thermal treatments do not guarantee the desired dipole orientation within the material matrix, as crystalline domains often tend to assemble into a low‐energy isotropic state. Therefore, heat‐treated organic piezoelectric materials are often subjected to additional tensile treatments (e.g., drawing) to achieve reorientation of crystalline domains. Subsequently, the crystallinity, the crystal structure, and the orientation of the crystal domains within the material matrix can be altered by the synergistic effect of thermal and tensile modulation.^[^ ^79^, ^194^, ^195^ ^]^ A one‐step thermal drawing of PVDF performed by Gomes et al.^[^ ^81^ ^]^ revealed that the fraction of *β*‐phase (*F_β_)* is positively correlated with the drawing ratio (*D*_r_) and negatively correlated with the drawing temperature (T). At *T* = 80 °C and *D*_r_ = 5, *F_β_* had a maximum value of ≈80%, accompanied by the highest availability of oriented [─CF_2_─CH_2_─] dipoles, and the strongest piezoelectric coefficient *d*_33_ = 34 pC N^−1^. Alternatively, Chen et al.^[^ ^178^ ^]^ reported a multi‐step method that processes the preheated PVDF membranes by melt drawing at varying *T* and *D*_r_, followed by a second annealing (Figure 7d). WAXD and FTIR revealed a *T‐* and *D*_r_‐dependent *β*‐phase formation, with an optimum content at *T* = 160 °C and *D*_r_ = 40 (*F_β_* = 96% and crystallinity of 58.8%) (Figure 7e). The Herman's orientational parameter for the *β*‐phase crystals along the melt drawing direction in the PVDF membranes produced under optimal drawing conditions reached ≈97%, aligning with SEM observations and small‐angle x‐ray scattering (SAXS) analysis (Figure 7f). Moreover, piezoelectric devices fabricated from the PVDF films showed high sensitivity for converting dynamic pressures into electric signals with excellent operational stability (Figure 7g).

Additionally, thermal drawing is important for dipole systems that show a limited response to electrical modulation. The drawing force directly initiates the reorientation of molecular chains/crystal domains along the drawing axis, thus guiding the rearrangement of dipoles constrained by inter‐ and intramolecular interactions. Crystal domain orientation (chain texturization) of PLLA is demonstrated as a key determinant of the macroscopic piezoelectric properties.^[^ ^102^ ^]^ Curry et al.^[^ ^196^ ^]^ performed a systematic evaluation of unidirectionally drawn PLLA membranes at *T* = 90 °C with *D*_r_ = 1 – 6.5. Compared to the weak noise collected from the pristine PLLA membrane, which exhibited random crystal domain orientation and low crystallinity, the PLLA drawn at *D*_r_ = 6.5 generated a voltage response of ≈0.9 V under 1.4 kPa impact forces. The Herman's orientation parameter increased gradually with *D*_r_, starting from 0 at *D*_r_ = 1, and reaching a maximum value of 0.89 at *D*_r_ = 4.6, followed by a slight decrease to 0.81 when *D*_r_ increased to 6.5. The crystallinity of the drawn PLLA exhibited a similar profile, the peak value of ≈55% occurred at *D*_r_ = 4.6. Similar crystallinity and orientation profiles as functions of *D*_r_ have also been demonstrated.^[^ ^97^, ^197^ ^]^ Accordingly, drawn PLLA films have been demonstrated to possess improved shear piezoelectric voltage coefficient *g*_14_ = 0.192 Vm N^−1^,^[^ ^197^ ^]^ and charge coefficient *d*_14_ = 5.2 pC N^−1^ (*D*_r_ = 4).^[^ ^97^ ^]^ By comparing the differential scanning calorimetry (DSC) profiles of PLLA films drawn at different Ts, Billimoria et al.^[^ ^198^ ^]^ revealed that the drawing of PLLA films at *T* > *T*_g_ can effectively orient PLLA chains and initiate the formation of fine crystals. However, when *T* exceeds *T*_g_ by ∼30 °C, thermally‐induced cold crystallization can increase brittleness, thus leading to premature rupture of PLLA during the drawing process.

Besides, in the case of piezoelectric biomaterials rich in hydrogen bonds, such as cellulose, the thermal treatments may substantially decompose the hydrogen bonding networks. Alternatively, the chain texturisation can be achieved by wet drawing.^[^ ^199^ ^]^ By varying the drawing ratio from 1.0 to 2.0, the transverse piezoelectricity *d*_31_ of cellulose‐based electroactive paper improved as the degree of alignment increased from 3.4 to 27.3 pCN^−1^.^[^ ^161^ ^]^

Thermal and tensile modulation is applicable to a wide range of dipole systems; however, drawing is primarily suited for thin films and may limit its utility in engineering dipoles within complex 3D scaffolds. Alternatively, by applying appropriate parameter settings, it is possible to leverage shear forces generated during extrusion‐based 3D printing to achieve the orientation of molecular chains and domains.^[^ ^200^, ^201^ ^]^ Relative to thermal stretching, extrusion‐based 3D printing has a wider range of applications and provides a reliable means of fabricating spatially complex structures. However, it must be investigated whether the molecular orientation during extrusion is sufficient to modulate the macroscopic piezoelectricity of the piezoelectric biomaterials.

### 4.3 Electrical and Tensile Modulation

**Figure 8:**
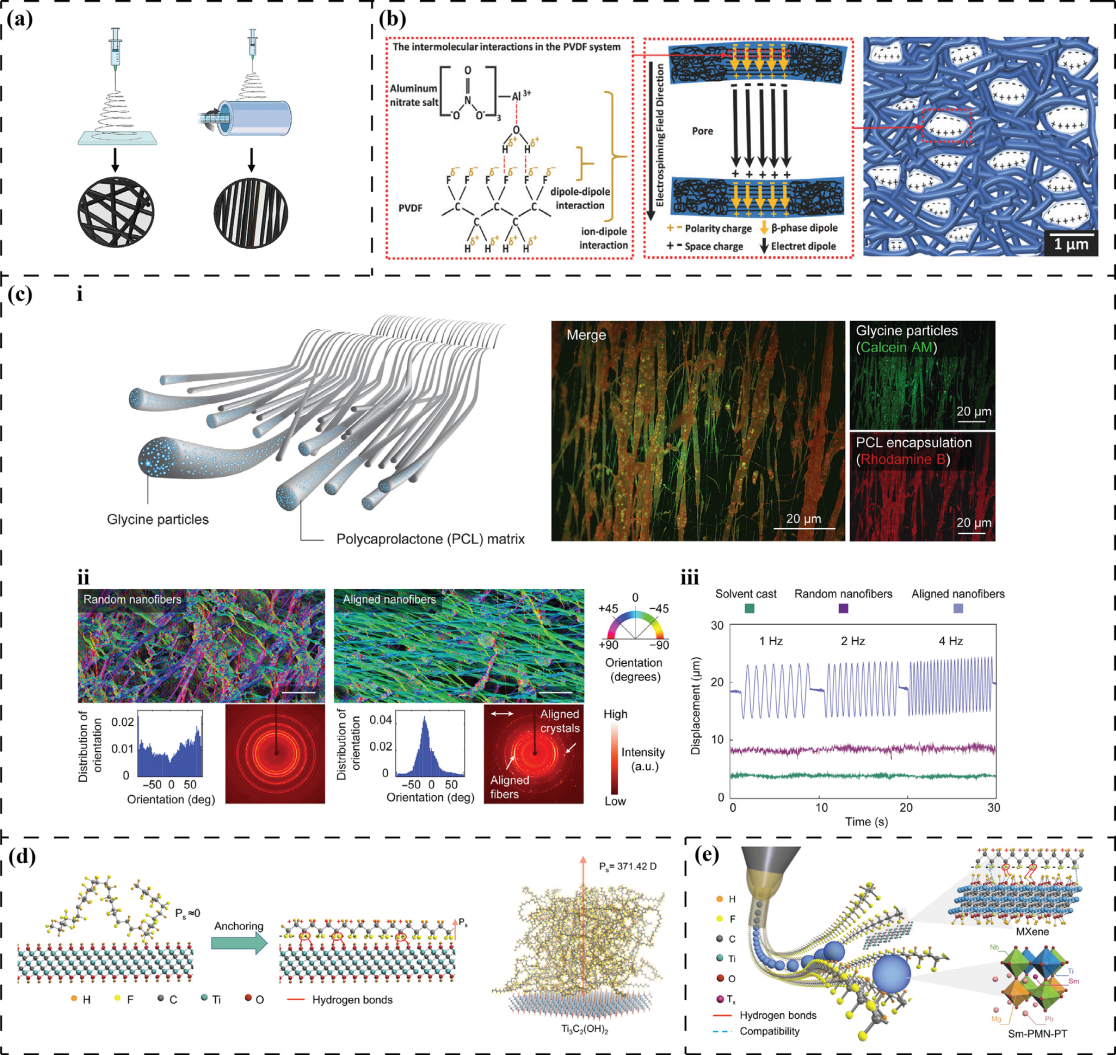
Electrospinning – the synergistic effect of electrical and tensile modulation on dipoles. a) Schematic illustration of the electrospinning setup. Reproduced under the terms of the CC BY 4.0 Licence.^[^ ^205^ ^]^ Copyright 2023, Springer Nature. b) Interaction between hydrated polar salts and [─CF_2_─CH_2_─] dipoles, space charges (piezoelectrets) between electrospun PVDF nanofibers, and the enhancement of piezoelectrets to the macroscopic piezoelectricity. Reproduced with permission.^[^ ^206^ ^]^ Copyright 2018, WILEY‐VCH Verlag GmbH & Co. KGaA. c_i_) Schematic illustration and fluorescent image; c_ii_) orientation colored SEM images, orientation distribution plots and 2D WAXS patterns; and c_iii_) displacement‐to‐voltage (20 V) responses of PCL/glycine nanofibers. Reproduced with permission.^[^ ^207^ ^]^ Copyright 2023, The American Association for the Advancement of Science. Effects of fillers on the dipole orientation. d) MD simulations of the anchoring of [─CF_2_─CH_2_─] dipoles to MXene surface, and the stacking of PVDF chains under electric field; e) Schematics of interaction between [─CF_2_─CH_2_─] dipoles and MXene during electrospinning process. Reproduced under the terms of the CC BY 4.0 Licence.^[^ ^208^ ^]^ Copyright 2022, Springer Nature.

Electrospinning is widely used as a fabrication method to produce nanofibrous meshes for tissue engineering applications.^[^ ^202^ ^]^ The high electrostatic force generated by the high voltage electrical field applied between the nozzle and the substrate collector overcomes the surface tension of the polymer solution, thereby stretching and accelerating the polymer solution to form nanofibers.^[^ ^203^ ^]^ Typically, electrospinning produces randomly oriented fibrous meshes. However, by changing the type and operation mode of the collector, it is feasible to modulate the orientation of the electrospun nanofiber.^[^ ^204^ ^]^ For example, unidirectional fiber alignment can be achieved by using a high‐speed rotating collector, i.e., the rotating drum provides traction to induce fiber organization (**Figure** **8**a).^[^ ^205^ ^]^

The modulation of dipoles provided by the high voltage electric field and ultra‐high D_r_ makes electrospinning a preferred tool for preparing piezoelectric products. In the case of PVDF, the key to modulating the [─CF_2_─CH_2_─] dipoles lies in inducing higher F*_β_*, enhancing crystallinity, and optimizing crystal domain orientation. Gee et al.^[^ ^209^ ^]^ statistically evaluated the contributions of solvent composition, flow rate, tip‐to‐collector distance, and electric field strength to F*_β_* in collected PVDF nanofibers, identifying their respective contributions as 63.3%, 21.8%, 9.94%, and 4.98%. Therefore, it appears that a rational solvent formulation is the most effective means to modulate *F_β_*. Yousry et al.^[^ ^206^ ^]^ added polar Al (NO_3_)_2_·9H_2_O salt to an 80/20 wt.% dimethylformamide/acetone solvent system to induce the formation of higher *F_β_* during electrospinning. The introduction of salt effectively suppressed the appearance of characteristic peaks of *α*‐phase, demonstrating that a higher *F_β_* was obtained. Moreover, the crystallographic planes of the *β*‐phase (200)/(100) aligned parallel to the collector substrate, signifying that the PVDF chains and [─CF_2_─CH_2_─] dipoles oriented along directions parallel and perpendicular to the film plane, respectively. The authors suggested that the increased *F_β_* arose from the molecular force interaction (i.e., hydrogen bonding) between the polar hydrated salts and [─CF_2_─CH_2_─] dipoles, facilitating dipole rotation during electrospinning. Additionally, the pores between PVDF nanofibers may form space charges, i.e., piezoelectrets (Figure 8b), thus contributing to the macroscopic piezoelectricity of the PVDF nanofibers. Consequently, the PVDF nanofibers with the addition of 10 wt% salt exhibited the optimal piezoelectric coefficient *d*_33_ = −116 pm V^−1^. The effect of solvent system, solvent concentration, tip voltage, working gap, collector type, ambient temperature, environmental humidity, and other factors to modulate the formation of the *β*‐phase in PVDF via electrospinning has been reviewed by Kalimuldina et al.,^[^ ^203^ ^]^ and He et al^[^ ^210^ ^]^ and will not be detailed further here.

Moreover, understanding the role of electrospinning in modulating other dipole systems is advancing. Curry et al.^[^ ^101^ ^]^ prepared PLLA nanofibers with different macroscopic orientations by varying the flow rate of the solution and the rotating speed of the drum collector. As the rotating speed increased from 300 to 4000 rpm, the Herman's orientation parameter of PLLA nanofibers gradually raised from ≈0.3 to ≈0.7 and plateaued, which coincided with the nanofiber arrangement observed by SEM. Moreover, the unidirectionally aligned PLLA nanofibers under high rotating speed (4000 rpm) exhibited an enhanced shear piezoelectric coefficient, *d*_14_ = 19 pC N^−1^, whilst randomly oriented nanofibers showed little‐to‐no charge when mechanically stimulated. However, the precise molecular mechanisms through which the coupled electrical and tensile forces modulate [─(C═O)─] dipoles within PLLA chains remain ambiguous. Additionally, Chorsi et al.^[^ ^207^ ^]^ prepared random and aligned electrospun polycaprolactone (PCL) nanofibers homogeneously embedded with ferroelectrically active glycine particles (Figure 8c_i_,c_ii_). When subjected to inverse piezoelectric actuation test (alternating 20 V electric field), highly oriented glycine‐PCL nanofibers (collected at 4000 rpm) exhibited clear displacement signals of ≈12 µm in amplitude, whereas neither randomly oriented nanofibers (collected at 0 rpm) nor solvent‐cast films exhibited any significant response. The longitudinal piezoelectric coefficient of aligned glycine‐PCL was further determined as *d*_33_ = 19 pC N^−1^, demonstrating the highest displacement‐to‐voltage response among all samples (Figure 8c_iii_). The stretching force and high voltage poling field in the electrospinning process were pivotal in reorienting glycine crystals and [─NH─C(═O)─] dipoles within the PCL matrix. Although further research is required to clarify the precise mechanism behind the reorientation of glycine crystals during electrospinning, this study offers valuable insights into scalable fabrication methods for glycine‐based piezoelectric materials. Despite its advantages in dipole modulation, electrospinning is primarily limited to producing thin films, which presents challenges for fabricating 3D piezoelectric scaffolds for BTE.

### 4.4 Filler Modulation

In addition to the modulation of dipoles by electric field, thermal treatment, and drawing, the incorporation of fillers is an alternative approach. The introduction of fillers with high piezoelectric activity into materials with weaker piezoelectric properties has been widely explored to enhance the overall piezoelectric performance of the composite.^[^ ^185^, ^211^, ^212^ ^]^ Alternatively, fillers with different surface chemistries and morphologies have been shown to interfere with the crystallisation process of piezoelectric materials.^[^ ^76^, ^213^, ^214^ ^]^

The chemical groups on the surface of fillers can interact with the dipoles of the piezoelectric material in different forms, directing the orientation of the dipoles and acting as nucleation points to induce the orderly stacking of molecular chains.^[^ ^215^, ^216^ ^]^ For example, Su et al.^[^ ^208^ ^]^ performed MD simulations of the anchoring mechanism of PVDF chains on a Ti_3_C_2_T_x_ MXene surface (Figure 8d). Hydroxyl groups on the MXene surface formed strong hydrogen bonds with F atoms in the [─CF_2_─CH_2_─] dipoles, inducing the self‐assembly of PVDF chains in a highly aligned TTTT configuration thus facilitating the formation of the ferroelectric *β*‐phase. Under the influence of an external electric field, these PVDF chains stacked along the MXene plane can exhibit a spontaneous polarization of up to 371.42 Debye. Moreover, during the electrospinning process, the intermolecular interactions between the [─CF_2_─CH_2_─] dipoles and MXene (Figure 8e) significantly enhanced the longitudinal piezoelectric coefficient of Pb_0.97_Sm_0.02_[(Mg_1/3_Nb_2/3_)_0.7_Ti_0.3_]O_3_ (SM‐PMN‐PT)/PVDF nanofibers. Specifically, fibers doped with 2.5 wt% MXene achieved *d*_33_ ≈ 26 pC N^−1^, an improvement over *d*_33_ ≈ 18 pC N^−1^ observed in pure SM‐PMN‐PT/PVDF nanofibers. However, excessive addition of MXene can lead to agglomeration, impeding the movement and orientation of PVDF chains and inhibiting *β*‐phase formation.^[^ ^208^ ^]^ Similarly, other studies report that excessive doping of fillers can negatively impact piezoelectric performance.^[^ ^217^, ^218^, ^219^ ^]^

Incorporating fillers with high aspect ratios has also proven effective in promoting crystallinity and regulating the formation of desirable crystalline phases. Vukomanović et al.^[^ ^220^ ^]^ conducted a comparative analysis of hydroxyapatite (HA) nanorods, BT nanospheres, BT nanosheets, BT micro blocks, and BT nanotextured rods (NTRs), evaluating their incorporation into PLLA. These fillers were selected based on the differences in their morphology and aspect ratio. Particle filled PLLA films were drawn (*D*_r_ = 5) at *T* = 90 °C to initiate crystallization. Thus, all particle filled PLLA films exhibited higher crystallinity than pure PLLA. Furthermore, high aspect ratio fillers, including HA (1wt.%) and BT NTRs (1wt.%) induced *β*‐phase formation, with BT NTRs achieving the highest crystal orientation. The voltage signal of the PLLA films, enhanced by at least 20‐fold under US stimulation, reflected the benefits of these fillers.

Additionally, for piezoelectric biomaterials that are difficult to prepare, in situ mineralisation of the material matrix provides an alternative method to modulate the piezoelectric properties. For example, Fang et al.^[^ ^221^ ^]^ achieved in situ mineralization of collagen fibrils through submersion in a strontium carbonate (SrCO_3_) mineralizing solution for 12 h. SrCO_3_ crystals gradually accumulated along the collagen fibril axis, from initial periodic banding to complete fibril filling. The mineralized collagen fibrils exhibited an enhanced longitudinal piezoelectric response (*d*_33_ up to 3.45 pm V^−1^) compared to both unmineralized collagen fibrils and pure SrCO_3_ crystals. This improvement was attributed to dipole reorientation during mineralization, although further investigation is needed to fully understand the underlying mechanism.

## 5 Piezoelectricity and Osteogenesis

Advances in integrating piezoelectricity into bone scaffolds are motivated by the natural piezoelectric properties of the native bone matrix, primarily composed of collagen and HA.^[^ ^20^, ^222^ ^]^ A fundamental advantage of piezoelectric scaffolds in BTE lies in their ability to spontaneously generate electrical stimuli upon mechanical activation, eliminating the need for invasive power sources.^[^ ^70^, ^223^ ^]^ These electrical stimuli are essential for scaffold‐cell interactions, promoting several cellular behaviors, including cell adhesion,^[^ ^28^, ^224^, ^225^, ^226^, ^227^ ^]^ cell proliferation,^[^ ^185^, ^186^, ^228^ ^]^ osteogenic differentiation,^[^ ^25^, ^29^, ^31^, ^192^, ^225^ ^]^ and biomineralization.^[^ ^188^, ^192^, ^229^, ^230^ ^]^ A summary of significant studies focusing on piezoelectric biomaterials for BTE is provided in **Tables** 1, 2, 3, 4, categorized based on the types of dipoles involved.

**Table 1:** A summary of studies of piezoelectric biomaterials with [─CF2─CH2─] dipoles in BTE applications.

Piezoelectric materials	Fabrication methods	Piezoelectric properties	External stimulation	Biological effects	Refs.
PVDF‐TrFE/BNNT film	Solvent casting annealing	d_31_* = 11 pmV^−1^ *ΔΦ* = 23–61 mV	1 Wcm^−2^ US 100 Hz burst rate 10 s/time, 2 time/day	In vitro (SaOS‐2 cells): US stimulated PVDF‐TrFE/BNNT film induced osteogenic differentiation (+).	[240]
PVDF‐TrFE film	Solvent casting thermal crystallisation contact DC poling	*d*_33_ = 0–20 pCN^−1^ *Φ* = ‐3–915 mV	∖	In vitro (MC3T3‐E1 cells): PVDF‐TrFE film (*d*_33_ = 15 pC/N, *Φ* = 391 mV) induced cell viability (+), osteogenic differentiation (+), fibronectin adsorption (+), integrin α_5_β_1_ expression (+).	[28]
PVDF‐TrFE film	Solvent casting Annealing corona poling	*d*_33_(L) = 10 pCN^−1^ *d*_33_(H) = 20 pCN^−1^ *Φ*_zeta_(L) = −53 mV *Φ*_zeta_(H) = −78 mV	∖	In vitro (BM‐MSCs): Group L film induced cytoskeleton expansion (+), osteogenic differentiation (+). In vivo (SD rats): Group L film induced complete calvarial regeneration.	[192]
PVDF‐TrFE/BGM film	Solvent casting annealing corona poling	*d*_33_ = 4.4 pCN^−1^ *Φ*_zeta_ = ‐40 mV	∖	In vitro (mouse BMSCs): Poled PVDF‐TrFE/BGM film induced cell proliferation (+), osteogenic differentiation (+), immunostaining intensity of CaSR (+). In vivo (SD rats): Poled PVDF‐TrFE/BGM film induced new bone formation (+).	[229]
PVDF‐TrFE/CFO film	Solvent casting thermo‐treatment stripe‐patterned DC poling	*d_33_* = 44.5–178.9 pm V^−1^ (200 µm wide stripe)	∖	In vitro (BMSCs): Surface potential gradient induced localised distribution of integrin α_5_β_1_, cytoskeleton orientation (+), osteogenic differentiation (+), mineralisation (+). In vivo (SD rats): Surface potential gradient induced regenerated bone volume (+).	[225]
PVDF‐TrFE film	Solvent casting, thermal crystallisation DC field poling	*d_33_* = 15 pCN^−1^ *Φ*(−) = −846 mV *Φ*(+) = 1200 mV	∖	In vitro (RAW 264.7 cells): Negatively poled surface induced anti‐inflammatory M2 expression (+); positively/negatively poled surfaces induced osteogenic differentiation (+), mineralisation (+).	[241]
PVDF/PMMA/PEI porous composite	Solvent casting vapour‐induced phase separation	*d*(30N @5 Hz) = 4.6 mV N^−1^	Oscillation 5 min/day	In vitro (SBF): Periodic loading induced CaP deposition. In vitro (MG‐63): PVDF/PMMA/PEI scaffold with dynamic oscillation induced osteogenic differentiation (+), biomineralisation (+). In vivo (SD rats): PVDF/PMMA/PEI scaffold induced osteointegration (+), recovered mechanical strength (+), osteogenic differentiation (+).	[242]

*Abbreviations*: PVDF‐TrFE – Poly(vinylidene fluoride‐co‐trifluoroethylene); BMSC – Bone marrow stem cells; BNNT – Boron nitride nanotubes; BGM – Bioactive glasses micro‐nano particles; CFO – CoFe_2_O_4_; DC – Direct current; US – ultrasound.

*Abbreviations*: PVDF – Polyvinylidene fluoride; PMMA – Poly(methyl methacrylate); PEI – Polyetherimide; SBF – Simulated body fluid.

**Table 2:** A summary of studies of piezoelectric biomaterials with [─(C═O)─] dipoles and other dipoles (if specified) in BTE applications.

Piezoelectric materials	Fabrication methods	Piezoelectric properties	External stimulation	Biological effects	Refs.
PLLA/PANI/CNT nanofibers	Electrospinning PANI/CNT deposition	*d = 0.57 mVN^−1^*	0.8 Wcm*^−1^* US 100 Hz burst rate 10 s/time 3 time/day	In vitro (human BM‐MSCs): US stimulated PLLA/PANI/CNT nanofibers induced osteogenic differentiation (+).	[29]
PLLA/collagen 3‐layered structure	Electrospinning Collagen adhesive layer	*d33 ≈ 2.95 pCN^−1^*	In vitro pressure 20 min/day	In vitro (rabbit ADSCs): Negatively/positively charged surfaces induced fibronectin adsorption by 2.7/2.1 folds.	[30]
PLLA nanofibers	Electrospinning contact DC poling	*d14(PLLA1.5) = 3 pCN^−1^* *d14(PLLA20) = 10 pCN^−1^*	Cell adhesion‐derived contractile force	In vitro (MSCs): Poled PLLA nanofibers induced intracellular Ca2+ fluorescence intensity (+), osteogenic differentiation (+), chondrogenic differentiation (‐); cell adhesion‐derived contractile force induced electrical potential on PLLA nanofibers; surface potential gradient induced integrated osteochondral differentiation.	[226]
PLLA/CMBT film	Solvent casting contact DC poling	*d*_33_(20% CMBT) = 3.5 pCN*^−1^* *Φ*_zeta_ = ‐50.45 mV	∖	In vitro (BMSCs): Poled PLLA/CMBT film induced calcium phosphate deposition (+), cell proliferation (+), osteogenic differentiation (+). In vitro (RAW 264.7 cells): Poled PLLA/CMBT film induced anti‐inflammatory M2 expression (+). In vivo (SD rats): Poled PLLA/CMBT film induced new bone formation (+).	[230]
PLLA nanofibers	Electrospinning annealing	*d*_14_(3000 rpm) ≈ 16 pCN*^−1^*	In vitro US at 40 kHz, 20 min/day In vivo US at 40 kHz, 30 min/day 5 day/week	In vitro (ADSCs): US stimulated PLLA (3000 rpm) nanofibers induced osteogenic differentiation (+), mineralisation (+). In vitro (BMSCs): US stimulated PLLA (3000 rpm) nanofibers induced osteogenic differentiation (+). In vivo (NSG mice): US stimulated PLLA (3000 rpm) nanofibers induced new bone formation (+).	[31]
PLLA nanofibers	Electrospinning	*Φ*(−) = 294 mV *Φ*(+) = 589 mV	∖	In vitro (MG‐63): PLLA (+) nanofibers induced better cell adhesion.	[243]
PHB film	Electrospinning diazonium modification	*d*_33_(PHB) = 2.5 pCN*^−1^* *d_33_* (PHB‐COOH) = 2.1 pCN*^−1^*	∖	In vitro (MC3T3‐E1 cells): diazonium modification induced proliferation (+);	[244]
Nylon‐11 nanoparticles ([─NH─C(═O)─] dipole)	Anti‐solvent methods	∖	US	In vitro (DPSCs): US/Nylon‐11 induced Ca^2+^ content (+), osteogenic differentiation (+).	[245]

*Abbreviations*: PLLA – Poly(l‐lactic acid); PANI – Polyaniline; CNT – Carbon nanotube; CMBT – Ca/Mn co‐doped BaTiO_3_.

*Abbreviations*: BT – BaTiO_3_; PHB – Polyhydroxybutyrate.

**Table 3:** A summary of studies of piezoelectric biomaterials with inorganic dipoles in BTE applications.

Piezoelectric materials	Fabrication methods	Piezoelectric properties	External stimulation	Biological effects	Refs.
BT‐coated Ti6Al4V disc (in vitro) scaffold (in vivo)	Electron beam melting (EBM) of Ti6Al4V BT surface modification	∖	LIPUS	In vitro (BMSCs): LIPUS stimulated BT‐coated disc induced cell area (+), vinculin fluorescence intensity (+), osteogenic differentiation (+), and cell apoptosis (‐). In vivo (New Zealand rabbits): LIPUS stimulated BT‐coated disc induced bone volume (+), bone penetration (+), osteointegration/bone‐scaffold binding strength (+).	[25]
BT‐coated Ti6Al4V disc (in vitro) scaffold (in vivo)	EBM printing of Ti6Al4V, BT surface modification corona poling	*d*_33_ = 0.7 pC N^−1^ *Φ =* −30.97 mV	Pressure at 0.1 Hz 1 h/day	In vitro (MSCs & HUVECs): Poled BT‐coated disc induced cell proliferation (+), osteogenic differentiation (+), migration (+), mineralisation (+), angiogenesis‐related gene expression (+), intracellular Ca^2+^ (+). In vivo (sheep): Poled BT‐coated disc mediated full bone/scaffold fusion (8 months), new bone formation (+), bone/scaffold integration (+), CaP deposition (+), vascularisation (+).	[187]
BT‐coated Ti6Al4V disc	TiO2 mediated BT coating electrical poling	*d*_33_ = 0.71 pC N*^−1^*	Periodic loading	In vitro (1.5SBF): Mechanical loaded BT‐coated disc induced CaP deposition (+), Ca/P ratio (+); negatively poled surface mediated Ca^2+^ (+).	^[^ ^188^ ^]^
BT/PMMA lamellar scaffold	Freeze‐drying PMMA negative pressure infiltration corona poling	*d*_33_(26.1 vol.% BT) = 7.5 pC/N *d*_33_(37.8 vol.% BT) = 20 pC/N *d*_33_(52.8 vol.% BT) = 39 pC N^−1^	∖	In vitro (rat osteoblasts): Increasing BT content induced cell proliferation (+), pseudopodia extension (+); moderate BT content (37.8 ∼ 42.6 vol%) induced highest cell orientation.	[246]
BT‐coated TC4 disk	BT surface modification electrical poling	*d*_33_ = 0.42 pC N^−1^	0.03 W/cm^2^ LIPUS 1 MHz 100 Hz pulse repetition 20 min/day	In vitro (MC3T3‐E1 cells): LIPUS stimulated BT‐coated disc induced cell proliferation (+), osteogenic differentiation (+), and intracellular Ca2+ concentration (+).	[189]
BT‐coated Ti6Al4V scaffold	EBM printing of Ti6Al4V MAO‐mediated BT coating	*I*(LIPUS) = 10–17.5 µA	0.03 W/cm^2^ LIPUS 1.5 MHz 1 kHz pulse repetition 20 min/day	In vitro (BMSCs): US stimulated BT‐coated disc induced cell adhesion (+), cell proliferation rate since day 4 (+), osteogenic differentiation (+), CaP deposition (+).	[227]
HA/BT scaffold	Core‐shell radial freeze‐casting corona poling	*d*_33_ *=* 2.4 pC N^−1^ *P*_r_ ≈ 0.79 µC cm^−2^	∖	In vivo (New Zealand rabbits): Poled HA/BT scaffold induced osteogenic differentiation (+), new bone formation (+), new bone penetration (+).	[26]
HAp/BTS disc	Sintering under pressure DC poling (1 kV/mm)	*d*_33_(50HAp‐50BTS) = 9 pC N^−1^	∖	In vitro (1.5SBF): Poled 50HAp‐50BTS stimulated the deposition of a stoichiometric HAp layer with 1.67 Ca/P ratio.	^[^ ^247^ ^]^
HA/BT scaffold	Digital light processing printing	*d*_33_(20HA‐80BT) = 2.15 pC N^−1^	0.03 W/cm^2^ LIPUS 1 MHz 50% duty 10 min/day	In vitro (DPSCs): Poled/LIPUS stimulated 20HA/80BT scaffolds induced cell morphology (+), osteogenic differentiation (+). In vitro (L929 cells): Poled/LIPUS stimulated 20HA/80BT scaffolds induced cell proliferation (+). In vitro (RAW 264.7 cells): Poled/LIPUS stimulated 20HA/80BT scaffolds induced M2 polarization (+).	[248]
PCL/BT lattice scaffold	FDM printing and corona poling (tip voltage 20 kV)	*d*_33_ *=* 0.1 pC N^−1^	∖	In vitro (SaOS‐2 cells): PCL/BT scaffold induced osteogenic differentiation (+).	[249]
Whitlockite pellets	Hydraulic pressing and annealing	*ΔV*(PWH‐750 NPs) = 473 mV	0.3 W cm^−2^ US at 1 MHz	In vitro (MC3T3‐E1 cells): LIPUS stimulated PWH pellets induced cell proliferation (+), osteogenic differentiation (+), CaP deposition (+), Piezo1/TRPV4 expression (+).	[27]

*Abbreviations*: LIPUS – Low intensity pulsed ultrasound

*Abbreviations*: HA/HAp – Hydroxy apatite; BTS – 80BaTiO_3_/20BaSO_4_; PCL – Polycaprolactone; MAO – Micro‐arc oxidation; FDM – Fused deposition modelling.

**Table 4:** A summary of studies of piezoelectric biomaterials combining organic and inorganic dipoles in BTE applications.

Piezoelectric materials	Fabrication methods	Piezoelectric properties	External stimulation	Biological effects	Refs.
PVDF/BT/Carbon scaffold	Polydopamine coating for PVDF high temperature carbonisation for BT@C nanoparticle Selective laser sintering (SLS) for scaffold high voltage poling	*ΔV*(1wt.%BT@C) = 5700 mV *P*_r_(1wt.%BT@C) ≈ 0.095 µCcm^−2^	0.8 W/cm^2^ US 100 Hz burst rate 10 s/time 3 time/day	In vitro (MG‐63 cells): US stimulated PVDF/BT@C(1wt.%) induced cell proliferation (+), osteogenic differentiation (+).	[186]
PLLA/BT/graphene porous scaffold	SLS corona poling	*ΔΦ*(PLLA/BT/graphene) = 1400 mV *ΔΦ*(PLLA) = 68 mV	0.8 W/cm^2^ US 40 kHz 100 Hz burst rate 20 s/time 3 time/day	In vitro (human UC‐MSCs): US stimulated PLLA/BT/graphene scaffold induced cell viability (+), osteogenic differentiation (+).	[185]

These studies predominantly focus on the impact of piezoelectricity on extracellular matrix (ECM) and cell behavior, with a focus on critical piezoelectric properties, namely piezoelectric coefficients and surface potential, and their role in various stages of osteogenesis. The studies can be generally divided into two scenarios: the regulation of the osteogenesis by 1) static parameters of piezoelectric biomaterials in the absence of external stimulation; and 2) dynamic piezoelectric response under external stimulation. Low‐intensity pulsed US (LIPUS) stimulation has gained considerable attention as a non‐invasive tool for evaluating piezoelectric scaffolds, both in vitro and in vivo. The Food and Drug Administration (FDA)‐approved LIPUS stimulation is painless, and the procedure requires no additional interventional devices. LIPUS has demonstrated significant therapeutic potential in bone healing, soft‐tissue regeneration, inflammation inhibition, neuromodulation, and dental treatments, as discussed by Jiang et al.^[^ ^231^ ^]^

Generally, LIPUS refers to the generation of 0.7 – 3 MHz wave form energy (intensity < 3 Wcm^−2^ at 100 Hz burst rate) from an US generating unit, introducing mechanical vibrations to target the scaffold surface.^[^ ^232^ ^]^ A typical LIPUS probe operating at 1.5 MHz emits an US beam with a radius of 11 mm and a propagation velocity of ≈1540 m s^−1^ in average tissue.^[^ ^233^ ^]^ According to the formula: *N* = *r* ^2^ *f*/*v*, where *N*, *r*, *f*, and *v* are the transition distance between near field and far field, radius, frequency, and travelling velocity of the US beams, respectively, the calculated *N* = 118 mm.^[^ ^234^ ^]^ Given that LIPUS beams must penetrate even the thickest soft tissues before reaching the bone, the previously calculated *N*, used as the radius of a circle, encompasses the mid‐thigh circumference of ≈95% of American adults.^[^ ^235^ ^]^ Thus, LIPUS can easily reach most bone tissue in clinical applications. Besides, piezoelectric materials are reported to possess reliable sensitivity for converting acoustic energy into electrical charge within the LIPUS frequency range.^[^ ^236^, ^237^, ^238^, ^239^ ^]^ Therefore, LIPUS provides a credible means of activating the piezoelectric response of piezoelectric scaffolds during both in vitro and in vivo evaluations.

### 5.1 Protein Adsorption

Most cells exhibit anchorage‐dependence and require a solid substrate or ECM to facilitate their typical behavior.^[^ ^250^ ^]^ Rather than directly binding to surfaces, cells adhere through interactions mediated by adhesion molecules and specific ligands. The protein quantity, type, and conformation on these surfaces are strongly influenced by the surface properties of biomaterials.^[^ ^251^ ^]^ Bone cells establish integrin‐ligand binding to fibronectin and vitronectin during the early stages of adhesion and migration.^[^ ^251^, ^252^ ^]^ The intra‐cytomembrane tails of the integrin connect to the actin cytoskeleton via several types of proteins, including *α*‐actinin, vinculin, talin, filamin, kindlin, and focal adhesion kinase (FAK), forming cell‐ECM focal adhesions.^[^ ^253^, ^254^ ^]^ The quality of such cell‐substrata attachment is crucial to maintain the cell morphology and facilitate the delivery of ECM‐cell signals.^[^ ^251^ ^]^ In this section, the effects of both the static properties of piezoelectric scaffolds and their dynamic piezoelectric response to external stimuli will be discussed, focusing on their role in protein adsorption.

**Figure 9:**
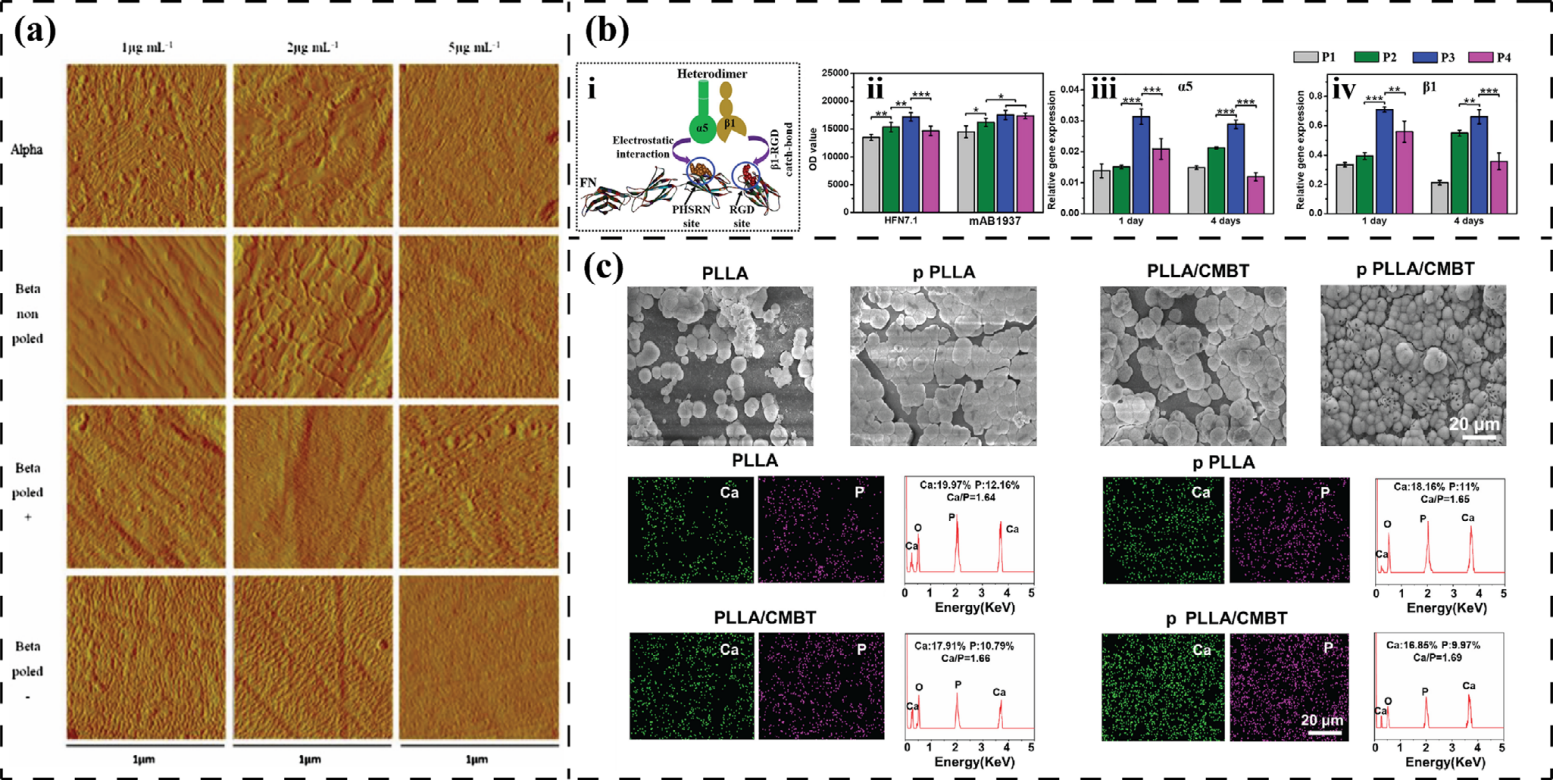
Piezoelectricity modulates the protein adsorption. a) AFM amplitude maps of fibronectin adsorption on the PVDF surfaces treated differently (FN concentration ranging from 1 to 5 µg mL^−1^). Reproduced with permission.^[^ ^255^ ^]^ Copyright 2012, IOP Publishing. b_i_) Schematic illustration of binding activity between fibronectin and integrins *α*_5_ and *β*_1_; b_ii_) fibronectin binding activity in PVDF‐TrFE films (P1, P2, P3, and P4) with varying surface potentials (−3, 106, 391, and 915 mV); gene expression analysis of b_iii_) integrin *α*_5_ and b_iv_) integrin *β*_1._ Reproduced with permission_._^[^ ^28^ ^]^ Copyright 2018, Elsevier. Modulating effect of piezoelectricity on calcium deposition. c) SEM images and EDS analysis of CaP deposited on negatively charged surfaces of PLLA, poled PLLA (pPLLA), PLLA/CMBT, and poled PLLA/CMBT (pPLLA/CMBT) films. Reproduced with permission.^[^ ^230^ ^]^ Copyright 2022, Elsevier.

The integration of piezoelectricity with osteoinductive/osteoconductive biomaterials enhances protein adsorption. For example, Ribeiro et al.^[^ ^255^ ^]^ found that the surface polarity of PVDF membrane significantly influences fibronectin adsorption, even without external stimulation. They studied *α*‐PVDF, non‐polar (unpoled) *β*‐PVDF, polar (negatively charged) −*β*‐PVDF, and polar (positively charged) +*β*‐PVDF surfaces with different concentrations (1, 2, and 5 µg mL^−1^) of fibronectin solutions. Limited fibronectin adsorption was observed on the *α*‐PVDF and non‐polar *β*‐PVDF surfaces at low concentrations (1 – 2 µg mL^−1^) (**Figure** **9**a). In contrast, a dense layer of fibronectin had formed on the surface of both −*β*‐PVDF and +*β*‐PVDF. Furthermore, at higher concentrations (5 µgmL^−1^), polar PVDF surfaces promoted an exponential increase in fibronectin adsorption as compared to non‐polar PVDF surfaces. Fibronectin adsorption was more pronounced for −*β*‐PVDF surfaces, enhancing the availability of binding sites (evaluated by fibronectin antibody HFN7.1) for MC3T3‐E1 cell adhesion.^[^ ^255^ ^]^ Alternatively, Liu et al.^[^ ^30^ ^]^ investigated fibronectin adsorption on the surfaces of negatively (−PLLA) and positively (+PLLA) charged piezoelectric PLLA nanofibers, and non‐piezoelectric PLLA nanofibers under the application of 80 kPa force. Protein analysis showed the enhancement of fibronectin adsorption on the charged surfaces of piezoelectric PLLA nanofibers under mechanical stimulation. The −PLLA surface exhibited ≈2.7‐fold higher fibronectin adsorption than the non‐piezoelectric PLLA, whilst +PLLA surface exhibited a comparable enhancement (≈2.1‐fold).

However, cell‐ECM binding activity does not exhibit an infinite positive correlation with the availability of binding sites. Specifically, the conformation of these binding sites, such as the spatial distance between the fibronectin peptide motif arginyl‐glycyl‐aspartic acid (RGD) and its synergistic site Pro–His–Ser–Arg–Asn (PHSRN), plays a crucial role. If the distance is too close/wide, it inhibits the effective adhesion between integrin and ligands.^[^ ^256^ ^]^ Tang et al.^[^ ^28^ ^]^ addressed the correlation between *Φ* (−3 to 915 mV) of positively charged PVDF‐TrFE films and the availability of RGD and PHSRN during fibronectin adsorption, evaluated by the primary monoclonal antibody HFN7.1 and mAb1937. ELISA readings indicated that the availability of both RGD and PHSRN was up‐regulated with the rise of *Φ*, and reached a peak at 391 mV. However, a further increase in *Φ* to 915 mV led to a slight down‐regulation (Figure 9b). The binding activity between fibronectin and MT3C3‐E1 cells (pre‐osteoblasts) on the PVDF‐TrFE films showed that the enhanced availability of RGD and PHSRN adhesion sites promoted *α*_5_ and *β*_1_ integrin gene expression. However, there was an abrupt decline when the *Φ* increased from 391 to 915 mV. This was attributed to a conformational change in *α*_5_ and *β*_1_ integrins accompanying the increasing *Φ*. Subsequent MD simulations suggested that increasing *Φ* enhances the exposure of RGD and PHSRN binding sites and reduces the RGD‐PHSRN distance, which reached an optimum value of 3.4 at 391 mV, ideal for effective integrin binding. Further elevation of *Φ* leads to an increase in the RGD‐PHSRN distance, thereby reducing binding activity. This finding underscores the importance of optimizing *Φ* to modulate cell‐material interactions, a critical consideration in designing scaffolds for piezoelectric BTE. However, there is limited research on the binding activity between adsorbed fibronectin and cells in response to dynamic charges generated by external stimuli.

### 5.2 Calcium Phosphate Deposition

HA, belonging to the calcium phosphate (CaP) family, plays a vital role in osteogenesis through mechanisms including HA crystal clustering, the formation of unorganised calcification nodules, and both interfibrillar and intrafibrillar mineralization.^[^ ^257^, ^258^ ^]^ Bone mineralization is dominated by the concentration of extracellular calcium ions (Ca^2 +^) and inorganic phosphate.^[^ ^259^ ^]^ The role of CaP in cell‐biomaterial interactions, fabrication approaches, and BTE applications has been comprehensively explored in numerous reviews.^[^ ^260^, ^261^ ^]^ The formation of the CaP layer promotes osteogenesis and osteointegration during implantation, and the rate of CaP formation is closely linked to the deposition of Ca^2+^ on the material surface.^[^ ^260^, ^262^, ^263^ ^]^

Negatively charged surfaces accumulate Ca^2+^, facilitating the subsequent electrostatic deposition of PO_4_^3−.[^ ^229^, ^230^ ^]^ For instance, Zhao et al.^[^ ^229^ ^]^ showed that the negatively charged poly(vinylidene fluoride‐trifluoroethylene) (PVFT) surface can attract higher amounts of Ca^2+^ releazed from bioactive glass microparticles (BGM) than the unpoled PVFT after 24‐h immersion in the same culture medium. Joo et al.^[^ ^264^ ^]^ reported that the incorporation of HAp fillers synergistically enhanced both electronegativity and piezoelectric coefficient of PVDF‐TrFE films, resulting in approximately a 40% increase in calcium deposition by osteoblasts after 14 days of culture on HAp/P(VDF‐TrFE) films (*d*_33_ ≈ 13 pC/N, *Φ* ≈ −390 mV), compared to pristine P(VDF‐TrFE) films (*d*_33_ ≈ 8 pC/N, *Φ* ≈ −240 mV). Additionally, Zheng et al.^[^ ^230^ ^]^ evaluated the CaP deposition on negatively charged PLLA films in their pristine states and PLLA films incorporating Ca/Mn co‐doped BT (CMBT) nanofibers (Figure 9c). The results demonstrated that both the incorporation of CMBT nanofibers and the poling process enhanced CaP deposition on PLLA surfaces, and the Ca/P ratio of deposited inorganic products on all surfaces was close to that of HA (CaP 1.67). Moreover, the CaP layer was shown to facilitate the adsorption of cell adhesion proteins, which are essential for the adhesion, proliferation, and differentiation of bone cells.^[^ ^262^, ^265^ ^]^ Furthermore, in a study conducted by Chernozem et al.,^[^ ^266^ ^]^ pre‐mineralised scaffolds seeded with MC3T3‐E1 cells exhibited superior cell adhesion and proliferation compared to pristine samples.

While piezoelectric substrates can promote mineralization, it is important to recognise that excessive inorganic phosphate concentrations in the ECM may induce vascular calcification. Nguyen et al.^[^ ^267^ ^]^ investigated the molecular mechanisms underlying the calcification of vascular smooth muscle cells (VSMCs) under high inorganic phosphate concentration. They demonstrated that elevated inorganic phosphate levels depolarize the membrane potential of VSMCs, activating voltage‐gated calcium channels (VGCC) and allowing excessive influx of Ca^2+^. The overloaded intracellular Ca^2+^ leads to oxidative stress and activates osteogenic differentiation, thereby accelerating the calcification of VSMCs, which constructs the tunica media of arteries and venules. Thus, determining the optimal piezoelectric parameters to regulate localised concentrations of Ca^2+^ and inorganic phosphate remains elusive. Further research should not only focus on promoting osteogenesis but also explore the interplay between mineral deposition and potential adverse factors that may impact other tissues, such as the vasculature.

### 5.3 Cell Fate Decision

New bone formation consists of several stages: condensation of mesenchymal stem cells (MSCs), differentiation into osteoprogenitors, proliferation of osteoprogenitors, osteoblastic differentiation and maturation, terminated differentiation into osteocytes, and mineralisation.^[^ ^268^, ^269^, ^270^ ^]^ A key attraction of using a piezoelectric bone scaffold is to re‐establish the endogenous bioelectric microenvironment. Thus, understanding the correlation between piezoelectricity and MSC fate is crucial. However, the fabrication of piezoelectric scaffolds for BTE is still in its initial stage. As mentioned in Section 4, various engineering techniques have demonstrated their effectiveness in modulating the dipoles within piezoelectric biomaterials, predominantly through thin films. These engineering strategies have been applied to create distinct piezoelectric properties within a single biomaterial system, allowing the impact of piezoelectricity on cell fate to be distinguished from the material's intrinsic bioactivity. Therefore, 2D piezoelectric bio‐platforms, including piezoelectric films^[^ ^29^, ^31^, ^192^, ^226^, ^240^, ^241^, ^271^ ^]^ and discs with piezoelectric coatings,^[^ ^189^ ^]^ have emerged as promising tools for assessing the specific role of piezoelectricity in directing osteogenic cell fate.

**Figure 10:**
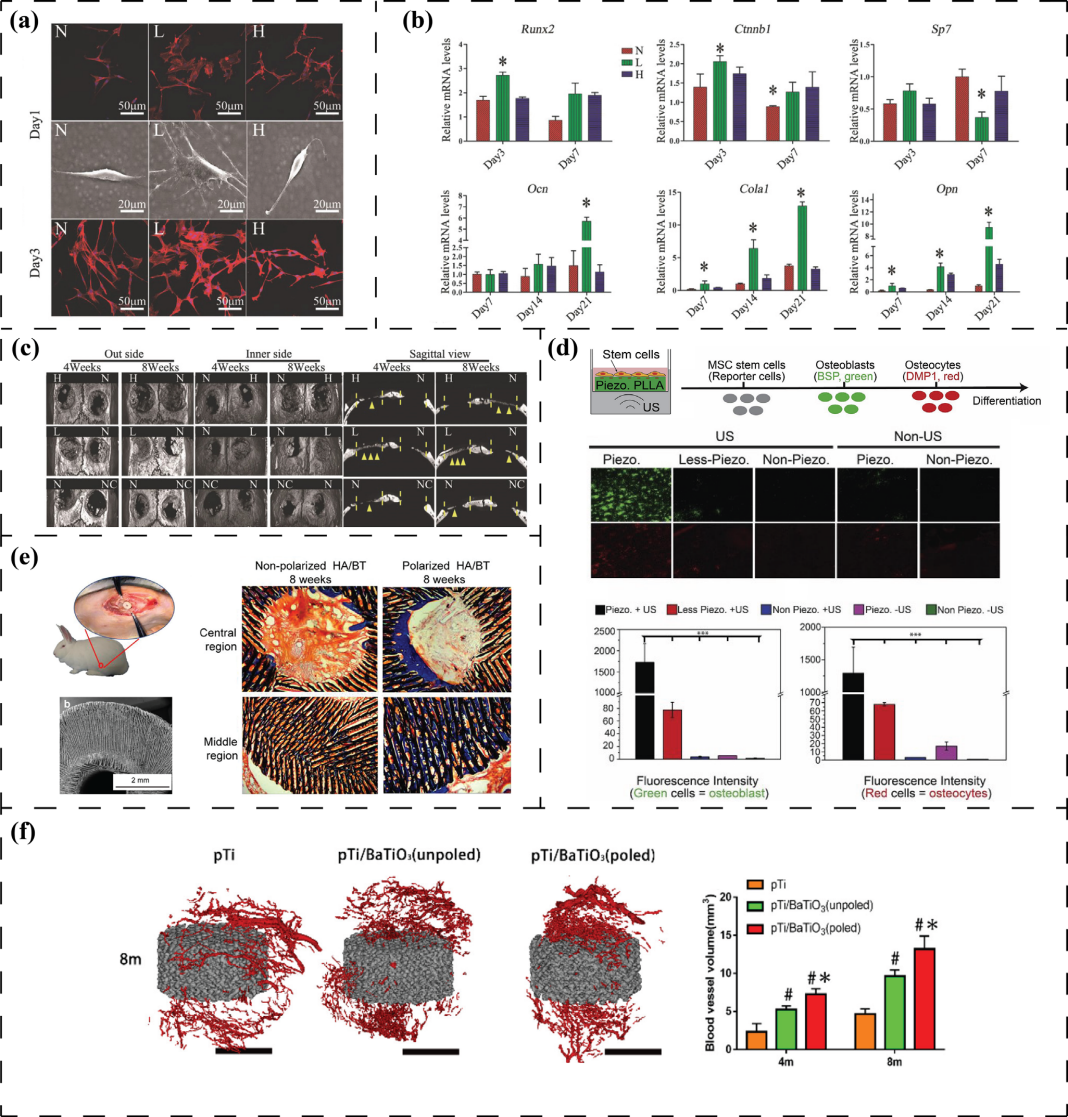
Effects of piezoelectricity on cell fate decision and different stages of bone regeneration. a) Immunofluorescence staining of the actin (red) and nucleus (blue) of BM‐MSCs cultured on the different group (N, L, and H) of PVDF‐TrFE membranes at day 1 and day 3. b) RT‐qPCR analysis of osteogenic differentiation‐related gene expression of BM‐MSCs during in vitro culture (up to 21 days). c) Micro‐CT images of harvested calvaria samples from SD rats during in vivo evaluation (N, L, and H denote the group of PVDF‐TrFE films, NC denotes the non‐covered negative control). Reproduced with permission.^[^ ^192^ ^]^ Copyright 2018, WILEY‐VCH Verlag GmbH & Co. KGaA. d) Schematic illustration of osteogenic differentiation of BMSCs cultured on piezoelectric PLLA scaffolds under dynamic US stimulation, and fluorescence images of bone sialoprotein (BSP, green) and dentin matrix protein (DMP1, red) after 3 days of culture. Reproduced with permission.^[^ ^31^ ^]^ Copyright 2020, Elsevier. e) Histological images of new bone formation (right) in the central/middle region of the unpoled/poled HA/BT scaffolds (bottom‐left) 8 weeks after implantation into femoral condyles of New Zealand rabbits (top‐left). Reproduced with permission.^[^ ^26^ ^]^ Copyright 2021, Springer Nature. f) Reconstructed 3D images of angiogenesis adjacent to porous Ti6Al4V scaffold, unpoled porous Ti6Al4V/BT scaffold and poled porous Ti6Al4V/BT scaffold 8 months after implantation into the vertebrae of sheep, and volumetric assessments of blood vessels adjacent to different scaffolds. Reproduced with permission.^[^ ^187^ ^]^ Copyright 2020, American Chemical Society.

In the absence of external stimuli, the modulation of cell fate by piezoelectric scaffolds is primarily influenced by their static properties, such as the piezoelectric coefficient and *Φ* (sometimes *Φ*_zeta_ is discussed instead). Zhang et al.^[^ ^192^ ^]^ investigated the effects of PVDF‐TrFE films with non‐poling (Group N, control), low *Φ*_zeta_ and *d*_33_ (Group L), and high *Φ*_zeta_ and *d*_33_ (Group H) on the osteogenic differentiation of rat bone marrow mesenchymal stem cells (BM‐MSCs) and their osteoinductive performance in a Sprague Dawley (SD) rat calvarial defect model. During the early stages of in vitro culture (day 1 and 3), all groups showed no significant difference in the growth of cell number. However, the BM‐MSCs on Group L films (*Φ*_zeta_ = −53 mV, *d*_33_ = 10 pC/N) showed a more spread, star‐like morphology (**Figure** **10**a), indicating the highest promotive effects on osteogenic differentiation. This observation was confirmed in an early (day 3) comparison of gene expression of osteogenic differentiation‐related runt‐related transcription factor (Runx2), an indicator of the initiation of osteogenesis (Figure 10b). Notably, in Group L, the expression of Sp7, a key regulator of mid‐to‐late‐stage osteoblast maturation, showed an earlier downregulation by day seven compared to Group N (*d*_33_ = 0 pC N^−1^) and H (*d*_33_ = 20 pC N^−1^). Furthermore, the late‐stage osteogenic markers osteocalcin (Ocn), collagen type I alpha 1 chain (Cola1), alkaline phosphatase (Alp), and osteopontin (Opn) were significantly upregulated on days 14 and 21. These findings suggest that modulating the piezoelectric coefficient and corresponding *Φ*_zeta_ of PVDF‐TrFE films can accelerate and enhance the osteogenic differentiation of BM‐MSCs. Moreover, varying degrees of bone regeneration were observed in the rat calvaria after 8 weeks, with complete and continuous regenerated bone present in the defect areas implanted with Group L films (Figure 10c). Therefore, the authors highlighted that the dose‐response correlation between piezoelectric properties and osteogenesis is not linearly positive.

In addition, the modulation of piezoelectric properties can influence the differentiation of stem cells to specific lineages, exemplified by the development of piezoelectric gradient scaffolds. For example, a PLLA nanofibrous membrane with a polarization gradient (*d*_14_ ranging from 3 to 10 pC/N) was developed by adjusting the poling field from 1.5 to 20 kV cm^−1^.^[^ ^226^ ^]^ MSCs seeded at the low polarization site (*d*_14_ = 3 pC N^−1^) exhibited a predominant chondrogenic differentiation profile (Sox‐9 and Col‐2) while osteogenesis was promoted at the higher piezoelectric value (Col‐1, Alp, Runx2 and Ocn).^[^ ^226^ ^]^ Subsequently, the fabrication of complex structures with functionalised piezoelectric gradients offers a promising strategy for bone interface tissue engineering applications (e.g., osteochondral, meniscus, tendons, and ligaments).

The incorporation of external mechanical stimuli, specifically through the combination of US and piezoelectric scaffolds, has been extensively validated in promoting osteogenesis. For example, osteoblast‐like Saos‐2 cells cultured on a piezoelectric PVDF‐TrFE/BNNT membrane exhibited more than a 1.4‐fold upregulation of Alp and Cola1 expression compared to the negative control, attributed to the application of 1 W/cm^2^ LIPUS over a 7‐day in vitro culture.^[^ ^240^ ^]^ Moreover, Sparc, an osteoblast‐specific marker indicating the inception of mineralisation and the assembly of collagen fibers,^[^ ^272^, ^273^ ^]^ was highly expressed (2.7 folds) due to the synergistic effect of increased piezoelectricity and applied US after 7 days.^[^ ^240^ ^]^ Apart from regulating osteoblast‐like cells, the introduction of US to piezoelectric scaffolds plays a crucial role in driving MSCs toward osteogenic lineage commitment. Das et al.^[^ ^31^ ^]^ investigated the effects of electrospun PLLA fibrous membranes collected at different rotational speeds: 3000‐rpm (piezoelectric), 1000‐rpm (less‐piezoelectric), and 300‐rpm (non‐piezoelectric), on the osteogenic differentiation of mice BMSCs and regeneration in a mouse calvarial bone defect model. Under static conditions without US treatment, bone sialoprotein (BSP) (reporter gene for osteoblasts) and BMP (reporter gene for osteocytes) expression in BMSCs was minimal in the non‐piezoelectric samples, indicating that while PLLA fibrous films exhibit good biocompatibility, their ability to induce osteogenic differentiation is limited (Figure 10d). An upregulation of BMP expression by nearly an order of magnitude in the piezoelectric samples indicated enhanced osteogenic differentiation, consistent with the findings reported by Zhang et al.^[^ ^192^ ^]^ However, when the piezoelectric samples were subjected to US treatment, the dynamically generated surface charges induced a substantial upregulation of BSP and BMP, increasing by nearly three orders of magnitude compared to the non‐piezoelectric samples under the same US treatment. Consistent with the in vitro findings, a mouse calvaria bone model demonstrated improved regeneration following 6 weeks of US treatment. Besides, Cai et al.^[^ ^189^ ^]^ reported that even a thin layer of BT coating with a low piezoelectric constant (*d*_33_ = 0.42 pC/N) on a medical TC4 disk could enhance the proliferation and osteogenic differentiation of MC3T3‐E1 cells with the aid of relatively low intensity LIPUS (0.03 W cm^−2^) stimulation. However, substantial variation in the ultrasonic parameters and piezoelectric material properties employed across studies highlights the need for more systematic investigations to optimize these combinations.

In addition to 2D bio‐platforms, growing interest is directed towards the development of biomimetic 3D piezoelectric scaffolds for bone regeneration. Damaraju et al.^[^ ^274^ ^]^ demonstrated that untreated 3D fibrous PVDF‐TrFE scaffolds with weak piezoelectricity (20 mV mm^−1^) exhibited predominantly chondrogenic differentiation‐related expression of MSCs under dynamic loading throughout a 28‐day in vitro culture. Alternatively, the annealed scaffold with pronounced piezoelectricity (1000 mV mm^−1^) promoted the strongest expression of osteogenic differentiation. According to Yang et al.,^[^ ^185^ ^]^ a PLLA/BT/graphene scaffold generated a high‐voltage electrical response 20 times greater than that of pristine PLLA scaffolds, resulting in the most robust proliferation and osteogenic differentiation‐related Alp activity in human umbilical cord‐derived mesenchymal stem cells (hUC‐MSCs) after 7 days of dynamic cultivation. In another work, Qi et al.^[^ ^186^ ^]^ immobilized carbon onto the surface of BT nanoparticles via polydopamine coating. The highest proliferation and Alp activity of osteosarcoma cell‐line MG‐63 cells were achieved by the BT@C‐1/PVDF (1 wt.% of carbon‐coated BT nanoparticles) scaffold with the strongest piezoelectric response under dynamic US stimulation.^[^ ^186^ ^]^ Apart from the in vitro assessment of 3D piezoelectric scaffolds, efforts have been made to bring these scaffolds to in vivo evaluation. When a 3D porous HA/BT scaffold was implanted into a defect on the femoral condyles of rabbits, it significantly enhanced new bone formation and bone tissue penetration in poled samples compared to their non‐poled counterparts, driven by the mechanical stimulation from the rabbits’ daily movement (Figure 10e).^[^ ^26^ ^]^

In general, 2D piezoelectric scaffolds can be fully infiltrated into the culture media, providing a homogeneous distribution of culture components as well as an intuitive view of the cellular behavior. Additionally, 2D piezoelectric bio‐platforms offer accessible methods to modulate and quantify their piezoelectric properties. Moreover, in vitro and in vivo studies based on 2D piezoelectric bio‐platforms have demonstrated the role of piezoelectricity in regulating the fate of MSCs and promoting osteogenic differentiation. In contrast, studies focused on osteogenesis and cell fate within 3D piezoelectric scaffolds remain limited, largely due to the scarcity of techniques available to characterize the localized piezoelectric properties across the surfaces of the 3D scaffold. Furthermore, 3D scaffolds face the intrinsic challenge of limited cellular and nutrient penetration deep within their structure, highlighting a major barrier in BTE, i.e., vascularization.^[^ ^275^, ^276^ ^]^

Bone tissue restoration is inextricably associated with complimentary vascular regeneration.^[^ ^277^ ^]^ The branched vascular network prevents ischemic necrosis by delivering sufficient oxygen, essential nutrients, and growth factors while efficiently removing waste.^[^ ^278^, ^279^ ^]^ This dynamic integration of osteogenesis and angiogenesis has driven the development of functional biomaterials targeting vascularised bone regeneration.^[^ ^275^, ^280^, ^281^, ^282^, ^283^ ^]^ For instance, Liu et al.^[^ ^187^ ^]^ reported that the electrically poled porous BT‐coated Ti6Al4V (pTi/BT) scaffold mediated higher expression of osteogenesis and angiogenesis‐related genes in MSCs and human umbilical vein endothelial cells (HUVECs), respectively, compared to the non‐poled pTi/BT and porous Ti6Al4V scaffold. In vivo studies further revealed abundant vascular aggregates forming on the pTi/BT surface 8 months after implantation into the vertebrae of a sheep (Figure 10f). In their following study, they reported that the poled BT/Ti scaffolds, combining cyclic loading, significantly promoted M2 polarization of RAW264.7 macrophages with enhanced expression of argnase‐1 (M2 marker), anti‐inflammatory IL‐10, and TGF‐β1, while poled BT/Ti scaffold with static culture conditions showed no significant difference from pristine Ti scaffold surface.^[^ ^284^ ^]^ The supernatants extracted from these macrophages cultured with poled BT/Ti scaffolds under dynamic loading conditions enhanced the Transwell migration of MC3T3 cells and significantly upregulated osteogenic gene expression. Moreover, both rat and sheep models, subjected to LIPUS stimulation for 2 weeks and natural movement simulation for up to 1 year, respectively, exhibited upregulated macrophage M2 polarization and enhanced subsequent bone regeneration with poled BT/Ti scaffolds. Their findings suggest that piezoelectricity inhibits the MAPK/JNK signalling pathway while activating the oxidative phosphorylation and synthesis of adenosine triphosphate in macrophages. The role of piezoelectricity in immunoregulation of osteogenesis is gradually being perceived, however, further exploration is required to understand the underlying mechanisms.^[^ ^241^, ^285^, ^286^ ^]^ Apart from the intrinsic potential of piezoelectricity, the presence of angiogenesis‐related ions in the additives might be an alternative approach to promote angiogenesis. Examples include ZnO,^[^ ^287^ ^]^ zinc‐whitlockite nanoparticles,^[^ ^288^ ^]^ and magnesium‐containing whitlockite nanoparticles.^[^ ^289^ ^]^ However, careful attention must be given to the concentration of these metal ions, as excessive levels carry a risk of cytotoxicity.^[^ ^290^ ^]^

### 5.4 Signalling Pathways

**Figure 11:**
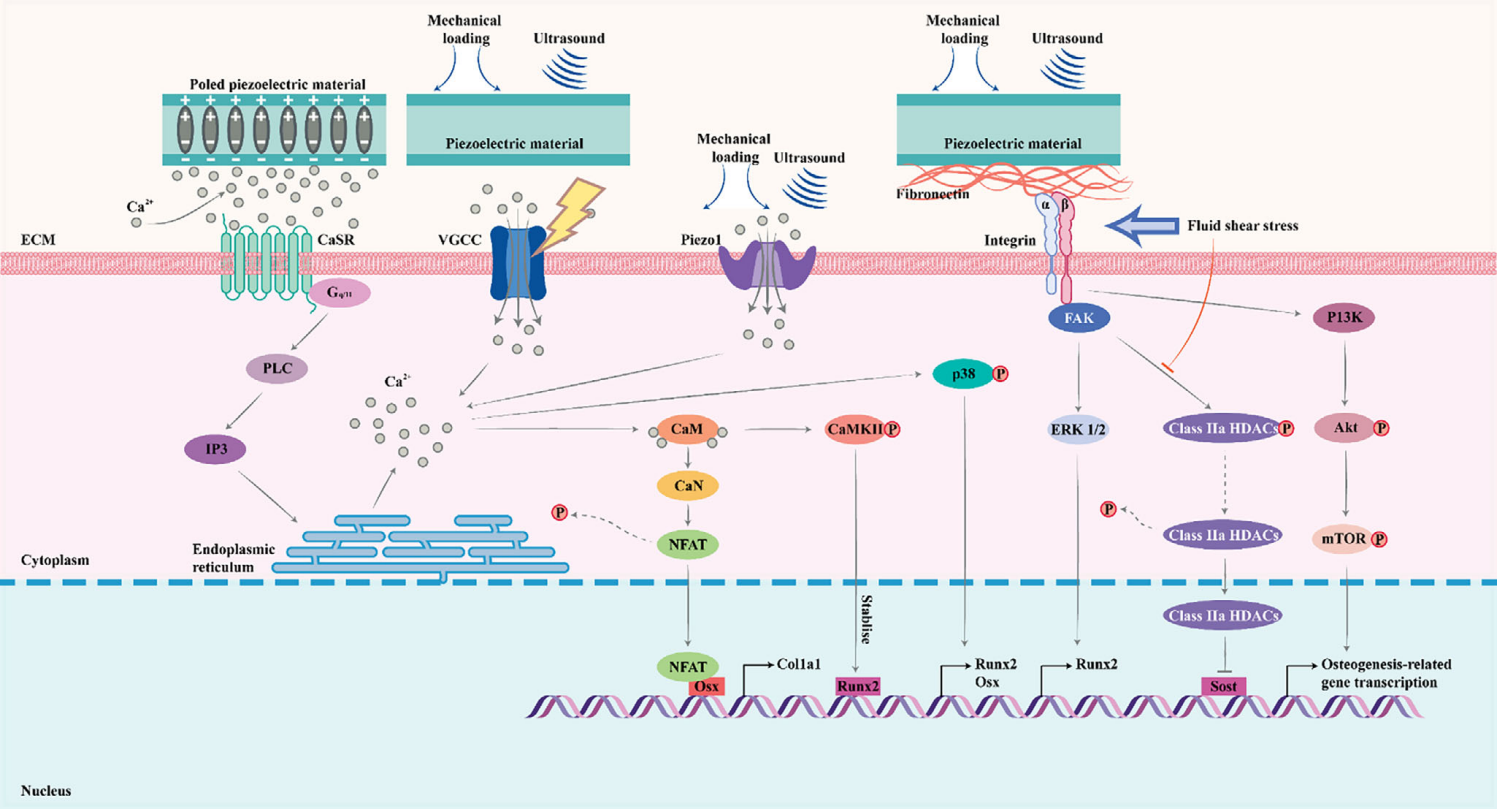
Schematic illustration of activated calcium‐related signalling pathways and mechanosensitive transduction pathways that promote osteogenesis in the presence of piezoelectric material and incorporated mechanical stimuli. Calcium‐sensing receptor (CaSR) can perceive the elevated Ca^2+^ concentration near a negatively charged piezoelectric material surface and activate phospholipase C (PLC) via the binding of heterotrimeric G‐protein G_q/11._^[^ ^294^ ^]^ Inositol‐1,4,5‐triphosphate (IP_3_), product of the hydrolysed intramembrane phosphatidylinositol‐4,5‐bisphosphate (PIP_2_) mediated by PLC enzyme, can bind to IP_3_R (IP_3_ receptor) and stimulate the release of Ca^2+^ from endoplasmic reticulum.^[^ ^294^ ^]^ The electrical charge generated on piezoelectric material surface under the stimulation of mechanical loading and ultrasound can trigger the depolarization of cytomembrane, leading to the influx of Ca^2+^ via opened voltage‐gated calcium channel (VGCC).^[^ ^32^, ^35^, ^189^ ^]^ The opening of mechanosensitive calcium channel Piezo1 can also contribute to the elevation of intracellular Ca^2+^ concentration when subjected to external mechanical stimulation.^[^ ^297^ ^]^ The increased intracellular Ca^2 +^ concentration can promote osteogenesis via calmodulin (CaM)/ calcineurin (CaN) pathway,^[^ ^298^ ^]^ CaM/CaM‐dependent kinase II (CaMKII) pathway, and p38 MAPK pathway,^[^ ^187^, ^299^, ^300^ ^]^ see text for details. Also, mechanical stimulation can activate mechanosensitive cascades, including integrin/FAK/extracellular signal‐regulated kinase (ERK1/2) pathway, that ultimately enhances the transcriptional activity of Runx2,^[^ ^301^, ^302^, ^303^ ^]^ while the FAK/class IIa histone deacetylases (HDACs) pathway can be interfered by fluid shear stress, resulting in the enhanced translocation activity of class IIa HDACs into the nucleus and the suppressed expression of sclerostin (Sost).^[^ ^304^ ^]^ Phosphoinositide 3‐kinase (PI3K)/Akt/mammalian target of rapamycin (mTOR) pathway is another integrin‐dependent pathway that can regulate the expression of several osteogenesis‐related markers.^[^ ^305^, ^306^ ^]^

During therapeutic piezoelectric stimulation, communication between cells and the extracellular environment is mediated by molecular receptors on the cytomembrane, that regulate downstream signalling cascades (**Figure** **11**).^[^ ^291^ ^]^ Several important signalling cascades related to bone healing processes have been identified.^[^ ^292^, ^293^ ^]^ Intracellular Ca^2+^, an important secondary messenger in stimulation‐responsive molecular cascades, plays a pivotal role in regulating osteogenesis.^[^ ^32^ ^]^ Intracellular Ca^2+^ concentration can be regulated by the static or dynamic surface charges generated by piezoelectric biomaterials through the activation of specific membrane receptors or ion channels. Zhao et al.^[^ ^229^ ^]^ reported a significantly enhanced calcium‐sensing receptor (CaSR) activity when culturing mouse BMSCs on the negatively charged PVFT (poled) surface, attributed to the accumulation of extracellular Ca^2+^ ions. The enhanced CaSR activity corresponded to a significant increase in the osteogenic differentiation gene expression of mouse BMSCs. Meanwhile, the use of NPS2143 (CaSR‐specific antagonist) resulted in a significant downregulation of osteogenic differentiation gene expression. CaSR is involved in the downstream G_q/11_/PLC‐IP_3_/endoplasmic reticulum (ER) signalling pathway, known as the store‐operated Ca^2+^ channel.^[^ ^294^ ^]^ This signalling cascade is initiated when IP_3_ binds to IP_3_R, prompting ER to release stored Ca^2+^ into the cytoplasm.^[^ ^294^, ^295^, ^296^ ^]^ Besides CaSR, the voltage‐gated calcium channel (VGCC) can be activated by the dynamic surface charge of a piezoelectric biomaterial excited by a mechanical stimulus, thus piezoelectric signals can directly regulate intracellular Ca^2+^ and its downstream osteogenesis‐related gene expression.^[^ ^32^, ^35^ ^]^ Such a dynamic surface charge initiates the depolarization of the cell membrane, allowing the influx of Ca^2+^. The role of VGCC was demonstrated through fluorescence imaging of intracellular Ca^2+^ in MC3T3‐E1 cells cultured on BT‐coated titanium alloy disks under LIPUS stimulation.^[^ ^189^ ^]^ However, treatment with verapamil, an L‐type VGCC inhibitor, resulted in negligible intracellular Ca^2+^ fluorescence, indicating the direct influence of piezoelectricity on VGCC.

Intracellular Ca^2+^ engages in various osteogenesis‐related signal transduction pathways, including the Ca^2+^/calmodulin/calcineurin/NFAT pathway.^[^ ^298^, ^307^ ^]^ Briefly, two pairs of Ca^2+^ bind to the intracellular calcium sensor calmodulin (CaM), exposing its hydrophobic surface. This surface binds to the catalytic subunit of calcineurin (CaN), initiating the conformational change of CaN and subsequently activating the phosphatase domain of CaN. The activated CaN dephosphorylates NFAT, exposing its nuclear localisation sequence and facilitating its translocation into the nucleus.^[^ ^298^, ^308^ ^]^ NFAT acts as a transcriptional factor and further causes NFAT‐dependent gene transcription. NFATc1 has been shown to enhance the transcriptional activity of osterix (Osx) and interplays with the canonical Wnt signalling pathway.^[^ ^309^, ^310^, ^311^ ^]^ The activation of the Wnt pathway elevates the transcriptional activity of Runx2. Runx2 is a fundamental regulator for the expression of many osteoblast differentiation marker genes, including Osx, Col1a1, BSP, Ocn, and Opn.^[^ ^269^, ^312^ ^]^ Moreover, the Ca^2 +^‐induced activation of CaM leads to phosphorylation of CaM‐dependent protein kinase II (CaMII), which plays an important role in modulating the stability of Runx2.^[^ ^313^ ^]^ Additionally, evidence shows that the phosphorylation of p38 mitogen‐activated protein kinase (MAPK) can be positively regulated by the concentration of intracellular Ca^2+^, whilst the activated p38 can target several osteogenesis‐related transcription genes such as Runx2 and Osx.^[^ ^187^, ^299^, ^300^ ^]^

It is worth noting that the stimuli themselves used to elicit piezoelectric response in the course of piezoelectric therapy may also lead to the regulation of osteogenesis‐related gene expression through specific signalling cascades. For instance, the Piezo1 mechanosensitive Ca^2+^ channel has been shown to serve as a pathway for the upregulation of intracellular Ca^2+^ in response to mechanical stimulation, such as cyclic loading and US.^[^ ^27^, ^314^, ^315^ ^]^ Moreover, in parallel, Piezo1 also facilitates lineage‐specific (e.g., MSCs, pre‐osteoblast MC3T3‐E1 cells, primary osteocytes) cell facilitation in osteogenic differentiation and bone formation via other signalling pathways, as systematically reviewed in the work of Qin et al.^[^ ^297^ ^]^ Apart from calcium‐related biochemical cascades, biomechanical stimuli, including mechanical strain and fluid shear stress (FSS), can be harnessed by specific molecular cascades to modulate the transcriptional activities of osteogenic differentiation factors.^[^ ^223^, ^305^, ^316^, ^317^ ^]^ As discussed earlier, integrins mediate cell‐ECM interactions and transmit biomechanical signals into intracellular pathways, such as the FAK/ERK(1/2)/Runx2, a highly investigated mechanosensitive pathway in MSCs that promotes osteogenesis.^[^ ^301^, ^302^, ^303^ ^]^ In osteoblasts, LIPUS stimulation alone increases integrin activity alongside Akt and p‐Akt levels, indicating activation of the phosphoinositide 3‐kinase (PI3K)/Akt pathway.^[^ ^305^ ^]^ Such molecular cascades can ultimately direct the upregulation of osteogenic differentiation markers, thereby enhancing osteogenesis.^[^ ^306^ ^]^ Evidence shows that the PI3K pathway in osteocytes can be initiated by FSS‐mediated activation of integrins.^[^ ^318^ ^]^ In parallel with the ERK(1/2)/Runx2 or PI3K/Akt pathway, extracellular FSS can transiently interfere with the FAK/histone deacetylases (HDACs) pathway via mechanosensitivity signalling. This interference reduces FAK‐mediated tyrosine phosphorylation of class II_a_ HDACs (HDAC4/5), facilitating their translocation into the nucleus.^[^ ^304^ ^]^ Class II_a_ HDACs have been shown to act as negative regulators of sclerostin (Sost) by modulating the occupancy of its upstream driving factor (MEF2C),^[^ ^319^ ^]^ and Sost possesses an inhibitory effect on the Wnt/*β*‐catenin signalling pathway, essential for stimulating osteogenesis and bone development.^[^ ^311^ ^]^

In general, signalling pathways are critical for understanding the correlation between piezoelectric response and cellular behavior. Inhibition of signalling pathways that are activated by mechanical stimuli can screen out cellular behavior that is specific only to the piezoelectric response. However, the investigation of signalling pathways in existing studies on piezoelectric scaffolds for BTE remains insufficient. Therefore, further research is necessary to elucidate the underlying mechanisms linking piezoelectricity to osteogenesis.

### 5.5 Summary

In view of the distinct cascades of events involved in bone regeneration, studies on the biological performance of piezoelectric biomaterials in the inflammatory phase and bone repair phase are evolving. The implantation of piezoelectric biomaterials into the bone defect site triggers immediate blood‐material interaction, i.e., plasma protein adsorption and inflammatory proceses.^[^ ^320^ ^]^ The enhanced surface potential amplitude and piezoelectric coefficients resulting from dipole alignment facilitate the adsorption of fibronectin, a glycoprotein that plays a crucial role in the adhesion of MSCs.^[^ ^255^ ^]^ However, this promotive effect follows a biphasic trend, i.e., an excessive surface potential amplitude (above ≈400 mV) will begin to suppress the binding availability.^[^ ^28^ ^]^ Moreover, piezoelectric biomaterials with *d*_33_ values in the range of 2 – 15 pC/N have been shown to effectively promote anti‐inflammatory M2 polarization of macrophages.^[^ ^230^, ^241^, ^248^, ^284^ ^]^ The introduction of external stimulation, which induces dynamic surface charges, can further enhance this immunomodulation.^[^ ^248^, ^284^ ^]^ The inflammatory phase has been widely appreciated as the initiating and decisive phase of bone regeneration, during which the recruitment of MSCs and M2 polarization‐mediated release of key growth factors such as TGF‐β1, BMP2, and VEGF lay the foundation for the subsequent osteogenic differentiation of MSCs, bone maturation, and vascularisation.^[^ ^321^, ^322^, ^323^ ^]^ Besides, a wide range of piezoelectric biomaterial systems with piezoelectric coefficient values between 0.1 to 20 pC N^−1^ under either static conditions or dynamic stimulation have been reported to enhance osteogenic differentiation, calcium phosphate deposition, and new bone formation in both in vitro and in vivo models (Tables 1, 2, 3, 4). However, studies corresponding to the bone remodelling phase, unfortunately, still lack clear progress.

## 6 Challenges and Future Perspectives

A crucial cornerstone for the advancement of piezoelectric biomaterials is the development of representative molecular models in the presence of complex environmental variables. Currently, the molecular models developed to explore the electromechanical response of organic piezoelectric biomaterials are predominantly consisting of single chains or simple stacking of multiple chains. Additionally, there is a lack of representations of electromechanical transitions of intra‐ and interchain dipoles in elevated dimensional models, as well as the coupling of highly oriented crystalline domains with amorphous domains or the consideration of water molecule infiltration. This representation is essential for probing the piezoelectric origins of specific biomaterials. The piezoelectricity of SF, for example, as discussed in section 2.2.4, may arise from the coupling effects of multiple dipole systems. In this context, developing MD simulations that account for interactions among multiple molecular systems,^[^ ^324^ ^]^ along with artificial intelligence‐driven modelling of dipole dynamics, might be the key in shifting piezoelectric biomaterial development from a trial‐and‐error approach toward on‐demand, molecular‐level material design. Furthermore, considering the wide application of US stimulated piezoelectric biomaterials, an effective microscopic finite element modelling of the interactions between ultrasonic waves and dipoles is required.

The prevailing methods for quantifying piezoelectric properties typically focus on the electromechanical response in isolation from external factors such as water and ions. However, in biological applications, the introduction of water molecules and free ions within bodily fluids can be fatal to the piezoelectric properties of biomaterials. For example, when CNC scaffolds are placed in an aqueous environment, the reorganisation of the hydrogen‐bonding network may result in disparate piezoelectric properties between the hydrated and anhydrous forms, which have not been discussed. Besides, existing dominant methods to characterize the piezoelectric response, including zeta potential measurement, open‐circuit voltage measurements, *d*_33_ piezometer, and PFM fall short in specific scenarios, such as evaluating piezoelectric responses on complex curved surfaces (e.g., 3D porous scaffolds) and dynamic surface charge in liquid environments. Although advanced technologies such as PFM offer nanoscale resolution and exceptional applicability for piezoelectric characterization, challenges remain when the technique is applied to bone‐related piezoelectric biomaterials and composite systems, particularly in isolating genuine piezoelectric responses from confounding artefacts such as electrostatic interactions.^[^ ^325^ ^]^ Excitingly, with the rapid advancement of machine learning across various fields, there have been attempts to train machine learning models capable of predicting functional properties (e.g., piezoelectric coefficients) based on key material characteristics (e.g., electrochemical, structural, compositional, and dimensional information).^[^ ^326^ ^]^ This approach undoubtedly offers a bottom‐up solution for evaluating piezoelectric biomaterials in circumstances where mechanistic studies are still in their infancy. However, the need for more advanced characterization technologies, such as in situ surface charge measurements in complex environments, is imminent.

In the context of BTE, particularly for addressing large bone defects, the utilization of 3D porous scaffolds is essential to overcome the limitations of spontaneous bone fracture‐healing in cases of extensive bone loss. However, current research on 3D porous piezoelectric scaffolds remains in its nascent stage and faces significant challenges due to the lack of engineering methods for modulating dipoles. As previously discussed, corona poling can direct the reorientation of dipoles within the material matrix to enhance piezoelectric properties. Nevertheless, while some studies have reported on the application of corona poling in 3D piezoelectric scaffolds, the impact of interconnected pores and the increased thickness of the piezoelectric material on the efficacy of corona poling remains unclear. Therefore, validating the effectiveness of existing methods in 3D porous piezoelectric scaffolds and exploring innovative approaches for dipole modulation are both imperative.

Any piezoelectric biomaterial requires robust and standardised in vitro and in vivo evaluation with and without external stimulation prior to clinical trials. An important prerequisite for in vivo evaluations, or even clinical trials, is that the in vitro model is adequately representative. However, it is not a facile task to evaluate the in vitro model, especially at the cell‐material interface using mechanical compression stimulation. Establishing representative compression set‐ups that accurately mimic the complex motions of animals and humans remains a formidable task. When US serves as the stimulation source, calibrating the efficiency of US propagation in in vitro and in vivo environments presents additional challenges. US propagation can vary in cell culture media and tissues due to differences in energy absorption capacities.^[^ ^327^ ^]^ Moreover, most US waves primarily reach the surface of the scaffold, undergoing scattering or conversion into other energy forms, rather than penetrating deep into the material.^[^ ^328^ ^]^ Therefore, careful design optimization of the porous structure in scaffolds is essential to ensure adequate US transmission and achieve a uniform distribution of piezoelectric response.

The long‐term performance of piezoelectric scaffolds for BTE under the demanding physiological environment remains an unresolved factor in evaluating their clinical translational potential. Efforts have been made to investigate the cell‐free degradation‐induced deterioration and long‐term stability of piezoelectric responses in piezoelectric scaffolds.^[^ ^101^, ^192^ ^]^ During long‐term dynamic mechanical loading in both in vitro and in vivo, changes in the scaffold structure due to degradation and potential loss of mechanical integrity may alter the spatial distribution, intensity, and ultimately failure of the piezoelectric response, a phenomenon that remains poorly understood. Furthermore, the influence of complex and dynamic interactions arising from both bioactivities at the scaffold surface and changes in the intrinsic properties of piezoelectric scaffolds during bone regeneration is still rarely discussed. For instance, whether calcium phosphate deposition on the scaffold surface interferes with the transmission of mechanical stimuli to the piezoelectric material or the subsequent delivery of piezoelectric signals to cells requires further investigation.

Finally, efforts should be directed toward identifying the most efficacious piezoelectric scaffold‐based therapy treatments and developing the comprehensive biosafety data that enables clinical translation and approval by regulatory authorities. For example, the optimal piezoelectric properties and stimulation processes that support distinct phases of osteogenesis. Additionally, the electrical activity of the piezoelectric scaffolds and the influence of external stimuli need to be evaluated with respect to the surrounding tissues to understand potential cytocompatibility issues. Furthermore, the role of incorporating bioactive factors and cells as an advanced combinatorial therapy requires further investigation.

## 7 Conclusion

This review emphasises the intrinsic molecular origins of piezoelectric phenomena in both inorganic and organic piezoelectric biomaterials, detailing the evolution of their microscopic dipole moments into macroscopic piezoelectricity. With the simultaneous advancement of characterization methods and molecular simulations, the understanding and manipulation of piezoelectric properties for biological applications are becoming increasingly explicit.

Attention is gradually shifting from materials with relatively simple mechanisms and significant piezoelectric responses (e.g., BT and PVDF), to materials with more complex mechanisms that still provide sufficient piezoelectric responses to meet specific application requirements, especially biocompatible and biodegradable piezoelectric biomaterials including PLLA, PHB, collagen, amino acid crystals, FF, and cellulose. Moreover, the exploration of mechanisms by which extrinsic factors induce pseudo‐piezoelectric responses, such as charged voids in piezoelectrets and diffusing ions in piezoionics is expanding the pool of candidate structures and biomaterials for piezo‐functionalised bone scaffolds. Dipole engineering via thermal, electrical, drawing, and emerging methods, including repulsive forces generated by the growth of ice crystals or highly ordered in situ crystallization driven by incorporated nanofillers, enables precise manipulation of piezoelectric properties. Consequently, the combination of advanced materials and processing techniques facilitates the engineering of specific performance criteria necessary for BTE.

Despite their potential, piezo‐functionalized bone scaffolds are still confronted with numerous challenges and remain in their infancy. Therefore, the development of new materials, more comprehensive molecular models, improved dipole engineering methods, advanced piezoelectric characterization techniques for complex structures, and systematic protocols is essential to unlock the intricate interplay between piezoelectric properties and cellular behavior. If this can be achieved, the credibility of the therapeutic efficacy of piezoelectric biomaterials in BTE will significantly improve, potentially accelerating their translation from laboratory research to clinical applications.

## Conflict of Interest

The authors declare no conflict of interest.
